# Statistical methods and modelling techniques for analysing hospital readmission of discharged psychiatric patients: a systematic literature review

**DOI:** 10.1186/s12888-016-1128-7

**Published:** 2016-11-18

**Authors:** Christoph Urach, Günther Zauner, Kristian Wahlbeck, Peija Haaramo, Niki Popper

**Affiliations:** 1 DWH Simulation Services, Neustiftgasse 57-59, 1070 Vienna, Austria; 2 Institute of Analysis and Scientific Computing, Vienna University of Technology, Wiedner Hauptstraße 8 – 10, 1040 Vienna, Austria; 3 National Institute for Health and Welfare, Mental Health Unit, P.O. Box 30, 00271 Helsinki, Finland; 4 Dexhelpp, Neustiftgasse 57-59, 1070 Vienna, Austria

**Keywords:** Psychiatric disorders, Readmission, Mathematical methods, Evaluation of research methods, Systematic review

## Abstract

**Background:**

Psychiatric services have undergone profound changes over the last decades. CEPHOS-LINK is an EU-funded study project with the aim to compare readmission of patients discharged with psychiatric diagnoses using a registry-based observational record linkage study design and to analyse differences in the findings for five different countries. A range of different approaches is available for analysis of the available data. Although there are some studies that compare selected methods for evaluating questions on readmission, there are to our knowledge no published systematic literature reviews on commonly used methods and their comparison. This work shall therefore provide an overview of the methods in use, their evolution throughout history and new developments which can further improve the research quality in this area.

**Methods:**

Based on systematic literature reviews realized in the course of the CEPHOS-LINK study, this work is a systematic evaluation of mathematical (statistical and modelling) methods used in studies examining psychiatric readmission. The starting point were 502 papers, of which 407 were analysed in detail; Methods used were assigned to one of five categories with subcategories and analysed accordingly. Our particular interest next to survival analysis and regression models is modelling and simulation.

**Results:**

As population sizes and follow-up times in the included studies varied widely, a range of methods was applied. Studies with bigger sample sizes conducted survival and regression analysis more often than studies with fewer patients did. These latter relied more on classical statistical tests (e.g. t-tests and Student Newman Keuls). Statistical strategies were often insufficiently described, posing a major problem for the evaluation. Almost all cases failed to provide and explanation of the rationale behind using certain methods.

**Conclusion:**

There is a discernible trend from classical parametric/nonparametric tests in older studies towards regression and survival analyses in more recent ones. Modelling and simulation were under-represented despite their high usability, as has been identified in other health applications and comparable research areas.

**Electronic supplementary material:**

The online version of this article (doi:10.1186/s12888-016-1128-7) contains supplementary material, which is available to authorized users.

## Background

Classic methods for evaluating studies in health care involve descriptive and test statistics, usually comparing two or more groups. This methodological pool is supplemented by various types of regression analysis, which also belong to the domain of classic statistics. A wide range of additional and sometimes innovative techniques have been developed more recently, not least thanks to evolving computer technology.

From the field of modelling and simulation, microsimulation models and agent-based models are increasingly being used in healthcare research [1], especially for decision analytic models or comparison of different intervention strategies [2]. The time-frame of recorded databases is naturally becoming longer, hence the value of methods for longitudinal data analysis and its research field is increasing [3]. As a further result, methods of survival analysis are being adapted and extended in order to meet the necessity of analysing recurrent events and handling gap-times [4, 5], multi-episode survival analysis [4] and more generally the increased utilization of epidemiologic methods [5].

The main advantage of such methods is their natural time-dependency, taking into account whether certain events must occur in sequence. Furthermore, they are more flexible than common statistical methods are with regard to input variables and information on a system level [6]. Simulation models on an individual level, technically often referred to as agent-based systems, look promising for this kind of analysis, which has – largely due to the required computational power – only emerged during the last 15 years [7]. They were originally developed in electrical engineering and informatics. Their main edge over classical statistics lies in their ability to describe not only correlations but also causal relationships. For example, agent based simulations allow modelling rules based on a variety of predictors that are known on a micro level (e.g. hospital data sets) be combined with knowledge about individual behaviour (e.g. regional service provider structures), permitting observation of emerging macroscopic behaviour. The information used can originate from different sources, which do not have to be linked, but can be integrated in the agent based simulation model. Results calculated under changed conditions (e.g. an altered hospital infrastructure) provide more insight on subsequent changes of readmission rates.

On the one hand, modelling and simulation aim to support an understanding of the effects of causal relationships on target values like readmission rates. On the other hand, more quantitative methods from the research field of machine learning have emerged in social sciences and have already been applied to similar research questions [8] as the ones addressed in this project. Yet, the requirements of these questions match some of the recent developments in this field, such as bootstrap methods for recurrent events [9]. All available methods can only be applied under certain conditions. Therefore, data must often be transformed while research on its impact [10] must be followed closely. Although some studies compare and analyse selected methods of a specific domain [11], we still lack an overall view on the mathematical and statistical methods used in this area of study.

The results of this study provide an overview on the methods used to analyse questions on readmission of psychiatric patients. This study was realised in the context of the EU funded CEPHOS-LINK project (FP7, project reference number 603264), therefore we will discuss the suitability of the methods identified in this studyon large ‘real world’ electronic health care registers. The methodological requirements for inquiries using this kind of real world data often differ from the classical statistical methods that are usually used for clinical trials. Simulation and decision analytic modelling have recently been widely used in the health care domain [12], as these methods are able to address some of these issues. It is therefore the aim of this review to find out which methods are used in studies with research questions on readmission of psychiatric patients, and to further ascertain whether these are suitable to our study project and how the methodological pool can be extended in order to obtain the most reliable results. Next to the assessment of currently used statistical methods, therefore, it needs to be asked whether dynamic modelling has the potential for analysing questions on readmission.

## Methods

In order to establish the current state of research in the field and to gather information for answering questions on readmission, comprehensive literature searches were first conducted in the electronic bibliographic databases Ovid Medline (ovidsp.ovid.com/), PsycINFO (hwww.apa.org/pubs/databases/psycinfo/), ProQuest Health Management (www.proquest.com/products-services/pq_health_management.html) and OpenGrey (www.opengrey.eu/). In addition, Google Scholar (scholar.google.com) was utilized. Relevant publications published between January 1990 and June 2014 were included. The full text was only reviewed from papers in English, German and Spanish.

Studies on predictors and determinants of readmission (rates) of psychiatric patients were searched using combinations of keywords (used as MeSH terms or free text, depending on the database), which describe mental disorders and readmission (for details, see the general description of the search terms in Additional file 1). Studies on readmission after discharge from psychiatric or general in-patient care with a primary psychiatric diagnosis were included. Admissions to day hospitals or community programmes were not considered to be readmissions.

Quantitative studies, including both observational and intervention studies, were selected for this review. Qualitative studies and case reports were excluded. Furthermore, all papers that did not include original data – e.g., editorials, letters to the editor, commentaries, reviews and meta-analyses – were not considered. Additional file 2 details the flow of articles through the selection process.

Studies that were not published as full reports were also excluded. Only studies examining adult populations (mean age ≥ 18 years) were included in the review, resulting in the exclusion of three studies. The study subjects had to be originally discharged from in-patient hospital care (psychiatric or general hospital care) with a main psychiatric diagnosis. The primary outcome of interest was readmission to in-patient hospital care; studies that did not report results on readmission were therefore also excluded. Due to the sheer number the detailed research question of each study is not presented here, but all of the included 407 studies either investigate which of their recorded predictors influence readmissions or focus on whether specific, previously defined variables affect readmission. Pairs of researchers (NP, CU, PP and GZ) independently reviewed all titles and abstracts of the publications retrieved from the databases and assessed the results of the searches. Strategies to deal with unclear or missing information were agreed upon in discussion groups. Subsequently, pairs of researchers screened full texts and extracted data independently. Following this careful selection process, 407 studies were eventually used for the analysis of statistical methods.

### Classification of the statistical methods

Classification was necessary in order to perform further applicable data extraction methods. Initially, all methods used in the 407 identified studies where extracted into tables with additional information on underlying data and general information on the investigated patients (for the full bibliography of the reviewed papers see Additional file 3). A quantitative overview was then generated, classifying the identified methods into five categories:

- Parametric tests
- Non-parametric tests
- Regression analysis
- Methods from survival analysis
- Dynamic models

These categories were chosen mainly in order to understand the vantage point from which the different studies looked at their data, ranging from classic testing of hypotheses (a and b), via the need to find and understand relationships (c), to event analysis (d) and dynamic models (e), which are not purely data driven but also incorporate structural knowledge. The classification is not disjoint. In most cases where survival analysis was performed, Cox-Regression models were also calculated, making the study also applicable for ‘regression analysis’ (c). However, we placed such studies into category (d) because Cox-Regression models belong to the methods dealing with the ‘time to event’, which is the classical domain of survival analysis. The following sections discuss each of the categories in more detail.

Parametric and non-parametric tests (the most frequently used were Mann-Whitney (32) followed by Wilcoxon (29), for a full list see Table 1) are classes of statistical hypothesis testing employed in order to decide whether previously defined groups are significantly different. Hence, data is usually obtained at one or two points in time. There are usually parametric as well as non-parametric methods for the same research question; data structure defines which test should be applied. However, it may be up to the researcher to decide whether to provide additional information about the underlying distribution or how the data is to be structured prior to testing.

**Table 1:** Parametric and non-parametric tests identified in the 407 studies on psychiatric readmission

Parametric Tests	
ANOVA (analysis of variance), Discriminant analysis, F-test, K means cluster analysis, Levene test, Likelihood ratio test, Linear discriminant analysis, MANOVA, Parc test, Principal component analysis, Power analysis, Pregibon Link test, Student-Newman-Keuls, T-test, Wald test, Z-test	
Non-Parametric Tests	
Chi-squared test, Discriminant function analysis, Fisher’s exact test, Friedman’s variance analysis, Kolmogorov-Smirnov test, Kruskal-Wallis test, Mann-Whitney U test, McNemar test, Tau test, Wilcoxon test	

Regression models are mostly used in order to find out which variables influence the outcome variable (in this case, readmission rates). Depending on the model, both the independent variable (e.g. diagnosis, medication) and the dependent variable (e.g. readmission) can change over time. Observation over longer periods of time is preferred. These models are therefore applied more for the purpose of exploration than for proving statistically significant differences within previously defined groups. The method applied corresponds to the type and structure of the input variables as well as the output. If the regression model is to be applied to count data like multiple psychiatric readmissions, Poisson regression may be the method of choice [13]. If the selected outcome is merely the occurrence of an event within a previously defined period of time [14], variable logistic regressions are usually employed. Both of these models are of interest because both show readmission. All regression models except COX models (which we classified as survival analysis) were assigned to this category for the purpose of evaluation of reviewed papers. However, the only other regression models to be mentioned directly were linear, Poisson and logistic regression. Many papers only referred to the models used as ‘regression’, making it impossible to perform a reliable, detailed classification.

Survival analysis provides a pool of methods which investigate the relationship of predictors to the time to an event; there is usually an initial event (e.g., discharge from a hospital) and a second event that can occur at any later point in time. Although data collection over a long period of time is preferred for these methods, they can also deal with censoring: when the measurements are not known for the full observation period. Survival analysis is the method of choice where the duration of time passed between the first and second event is of interest. The event of major interest in these studies is readmission to a hospital. One reason for looking at this category is to find out how many research groups analyse the time to readmission compared with those which use the binary outcome of readmission within a previously fixed amount of time. Another is that these methods originate from the field of epidemiology [5], where systematically collected data like in randomized controlled trials (RCTs) are a rarity. Survival analysis methods are therefore possibly more applicable to routinely collected reimbursement data than to hypothesis tests, which often assume a very strict setting on data acquisition. In addition, methods of survival analysis can deal with censoring problems, which may leave us with a greater number of data sets for evaluation.

Simulation and decision analytic models have recently been widely used in the health care domain. One of their main advantages is their natural time-dependency, taking into account whether certain events can only occur in sequence. Furthermore, they are more flexible than common statistical methods are with regard to input variables and information on a system level [6]. Simulation models on an individual level [7] as well as system behaviour modelling on a macroscopic level [12] look promising for this kind of analysis, which has only emerged during the last 15 years. Methods from this innovative research field are included in the last category.

## Results

**Fig. 1:**
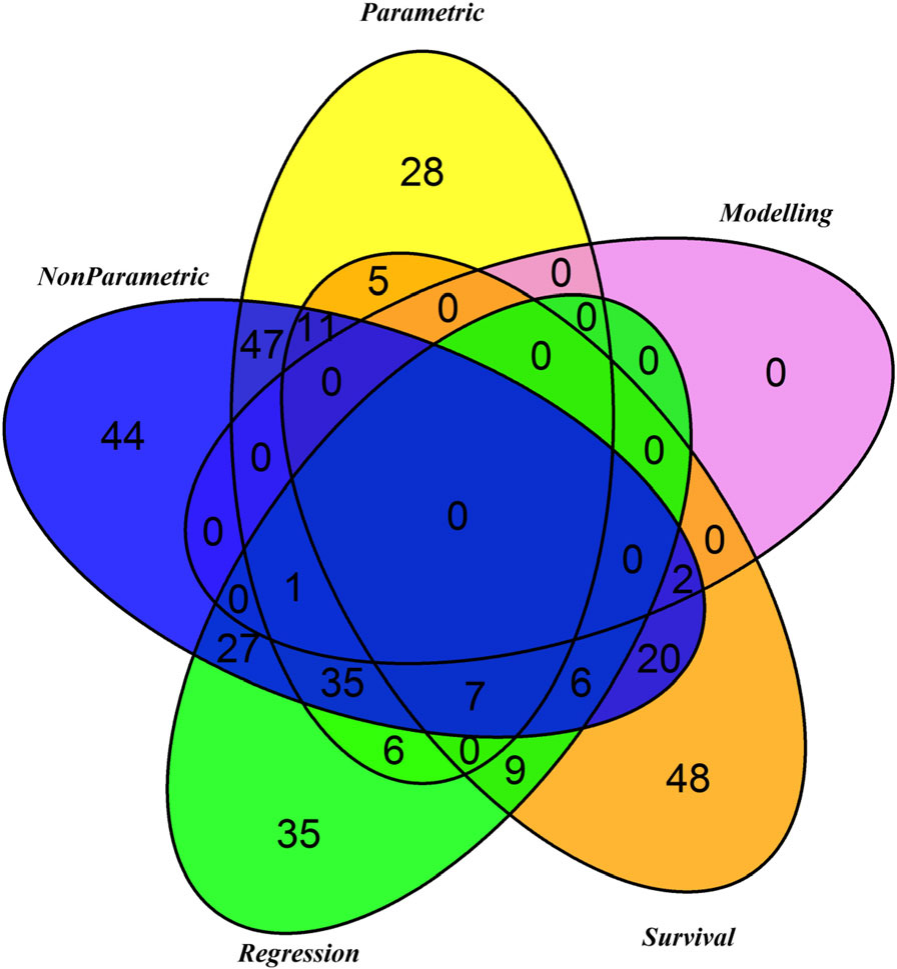
Venn diagram of identified methods used in studies on psychiatric readmission. Based on the systematic evaluation of the 407 papers included in the review on psychiatric readmission (years 1990–2014), the mathematical and statistical methods employed were divided into five sub-categories. The figure shows the number of studies using methods from several categories

The large number of studies reviewed (*N* = 407) guarantees a broad overview of methods used in the analysis of readmission of adults with mental illness in real world application. However, as most of the included studies did not focus primarily on the description of mathematical methods used, the rationale behind the decision for a given method was not included in many cases. Figure 1 gives an overview of the methods used in the reviewed studies.

### Population size in the studies

**Fig. 2:**
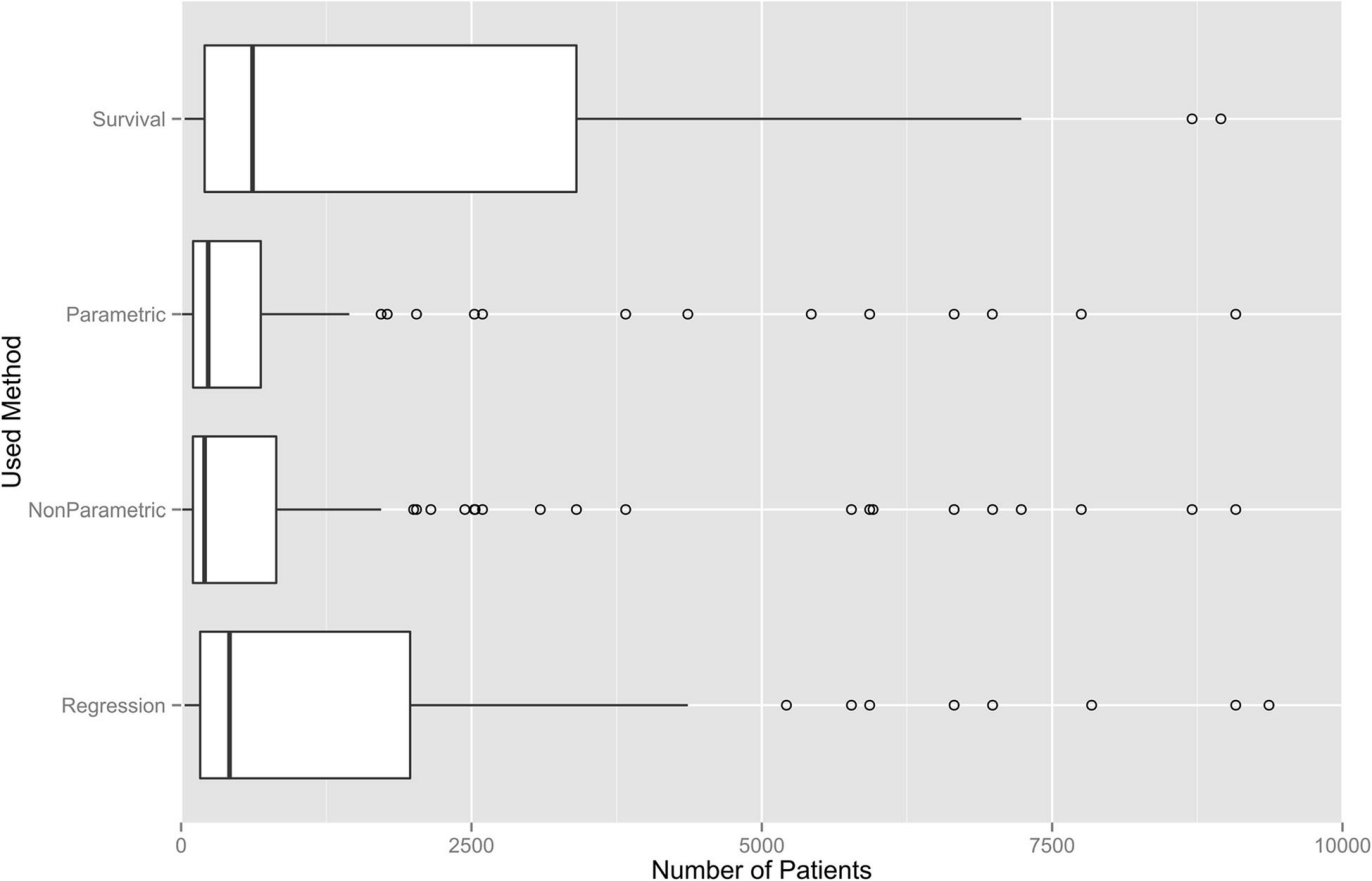
Boxplot of population size in relation to the methods used in the reviewed studies on psychiatric readmission. The methods used in the 407 papers included in the review (years 1990–2014) were divided in sub-categories representing different types of analysis

As the usability of statistical tests always depends on the population size of the data, we analysed the number of patients under observation. Studies with larger population sizes featured survival analysis (median 615) and regression models (median 417) more often than studies with smaller population sizes did. Parametric and non-parametric methods were performed with sample size medians of 233 and 202 respectively. The minimum of analysed patients in all papers was 7. Such a low number allows no relevant conclusions. The maximum were 408 158 patients (for the distributions of patient numbers and follow up times, see Fig. 2 and Additional file 4). It is interesting to note that regression and survival analysis was also reported in studies with quite low population numbers, although these methods do not yield reliable results under those circumstances.

### Analyses of follow-up time

Similar analyses as for population size were performed for the follow-up times reported in the studies. In contrast to the population size, where only a minimal number of documents did not report the number, follow-up time was subject to several problems. It was not possible to complete the data extraction sheet in detail for 103 studies, largely due to missing data, inconsistencies in reported follow-up times or unclear phrasing.

**Fig. 3:**
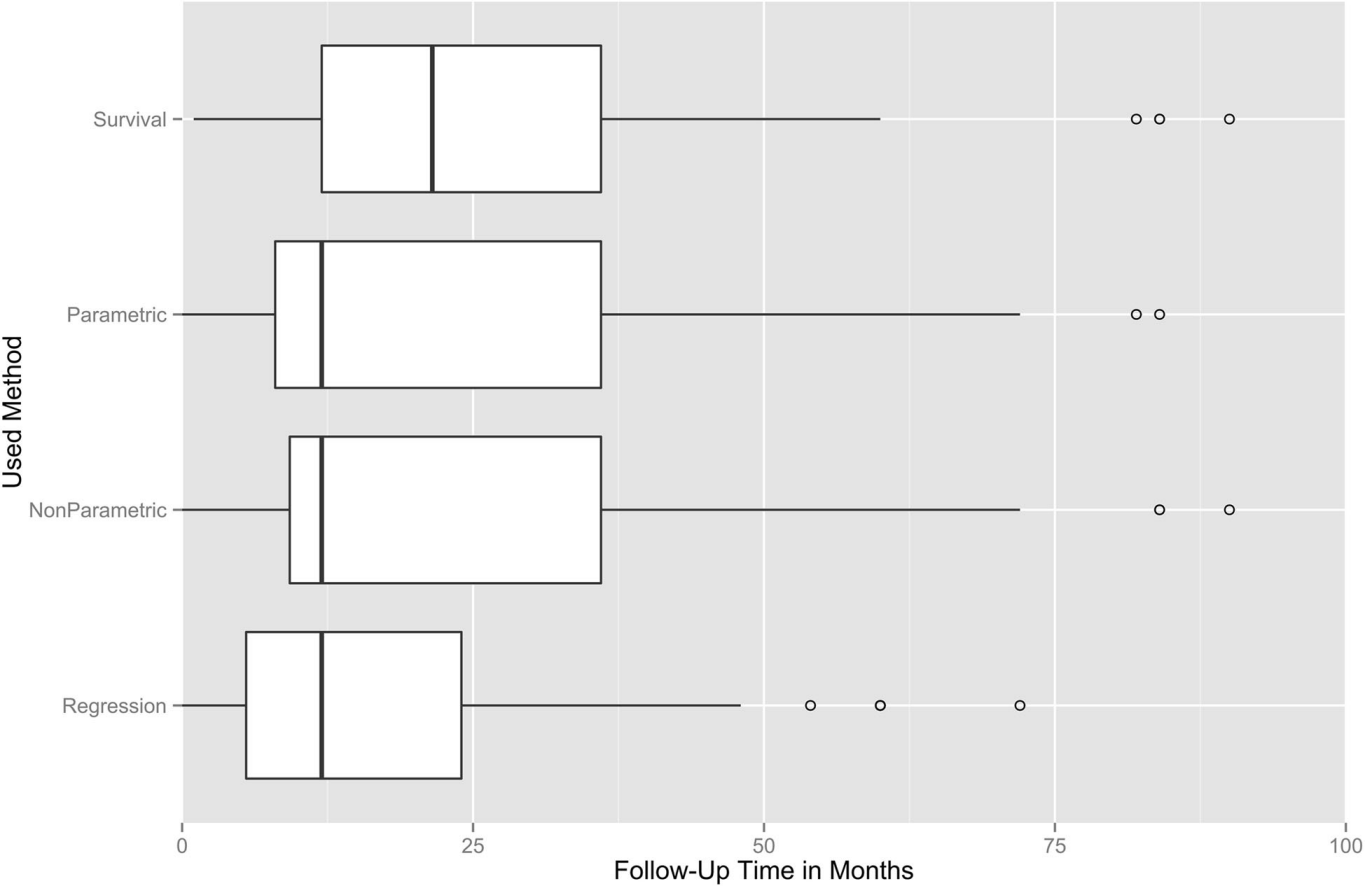
Boxplots of the follow-up times in months in the reviewed studies on psychiatric readmission. The methods used in the 407 papers included in the review (years 1990–2014) were divided in sub-categories representing different types of analysis. The figure shows the relation between the used category and the follow-up time

Overall, the median of follow-up times was 12 months for studies using regression, parametric and non-parametric methods, and 21.5 months for studies that included survival analysis. The boxplot in Fig. 3 shows the distributions among the four categories (for details also see Additional file 4).

### Used methods

All included studies used descriptive statistics and the majority (250 studies, 61%) used test-statistics like chi-square or Mantel-Haenzel. Cox proportional hazard models remain the dominant method for in-depth analysis of the point in time for readmission, because they can deal with both non-normally distributed and censored data [15].

A look at the overall number of methods used in the studies reveals quite clearly the dominance of chi-square analysis, with 159 (39%) out of the total of 407 studies using it, representing 39% of all studies. Out of the other methods, only the Mann-Whitney U test (32; 8%) and the Wilcoxon test (29; 7%) were also performed in more than 5% of all papers. Regarding studies reporting parametric tests, t-tests and Analysis of Variance are employed the most being used in 78 (59%) and 46 (35%) of those studies respectively.

A look at regression models used in the studies reveals a frequent failure to report the model in detail. Nevertheless, the dominant method is logistic regression, which is used in 32% (129) of all publications. In contrast, multiple linear regression is only used in 12 (2.9%) and binomial/Poisson regression in 11 papers (2.7%) of the reviewed studies. Also, the variables which are included in the regression models are spread very inhomogeneously across the papers depending on the kind of predictors the study aims at. However, most commonly included regressors are age, gender, length of stay and diagnosis.

Beyond the classical descriptive analysis of the methods used, it is of great interest how different statistical methods or modelling and simulation with statistics are combined in order to answer a research question. A Venn diagram (Fig. 1) was chosen for the visualization of the outcomes of the literature research. The diagram shows that quite a high number of studies use or at least describe only one method or classified group. Modelling and simulation is used only in three publications.

Overall, a detailed review of the methods used reveals that a vast number of different methods were employed. Table 1 shows a detailed list of the parametric and non-parametric tests identified in the studies. As several documents merely list the methods in the result tables or after usage in the discussion section, they are often not explained in detail. This may result in some fuzziness. Nevertheless, the overall information on the distribution of used methods remains stable.

**Fig. 4:**
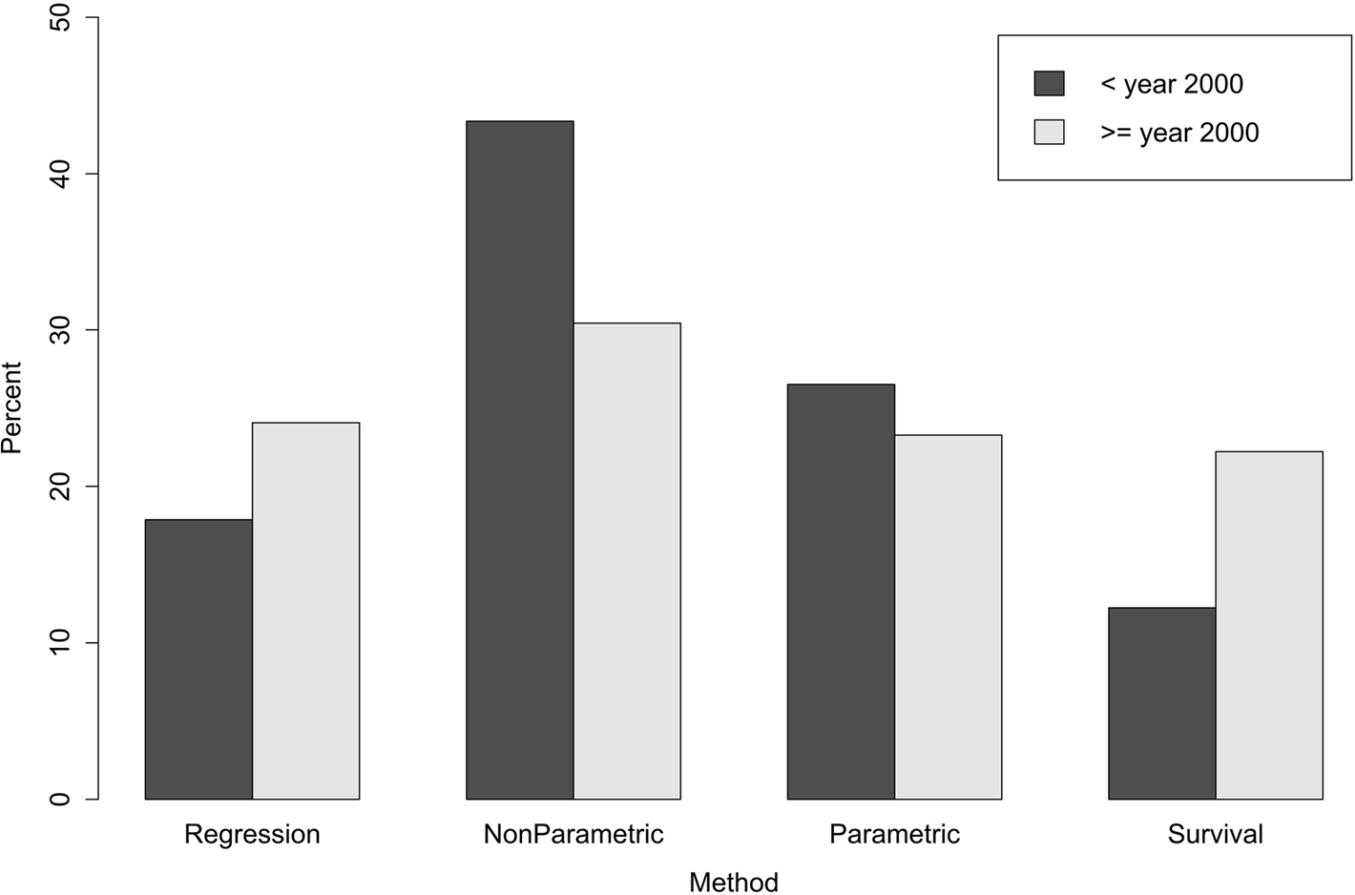
Total percentage of studies on psychiatric readmission published before or after 2000 out of studies published between 1990 and 2014. 407 studies were separated into those published up to 1999 and those published in 2000 and later

The bar plot in Fig. 4 represents the number of studies that used at least one method from one of the defined categories (accordingly, it was possible for each study to be classified into more than one category) by year of release as a percentage of all studies published during the defined time period. The dark grey data shows studies published in 1999 or earlier (altogether 196 studies) and the light grey data represents methods used in the year 2000 or later (378 studies).

## Discussion

We conducted a review of the methods used for the analysis of readmission of psychiatric patients. We identified, classified and assessed the methods found by year of publication, study cohort size and follow-up time.

The quality of the reviewed papers varied widely, which further complicated the extraction of statistical methods employed. Sufficient information for a reproduction of the study was frequently not available; this included the absence of an exact definition of the study population, or information on how the variable selection for the models was processed. Many studies categorized or dichotomized variables, often resulting in a loss of information (see [10, 16] for an analysis of the problem), without offering an explanation for doing so. In some cases, we were able to assume that the reason for doing so was the method of analysis, in other cases the decision seemed quite random. One general point of criticism is that a high number of publications did not report their methods in detail, resulting in inexplicable solution strategies and limiting the type of detailed classification intended in this paper. A more fine-grained model categorisation is frequently absent in the case of regression models, making it impossible to understand how to make an exact interpretation of the statistical results and identify the assumptions employed in the models.

The task of choosing the best data evaluation strategy is strongly connected to other framework conditions in the CEPHOS-LINK study, especially the register-based longitudinal data from different European countries and the individual characteristics of their healthcare system and data reporting. Country-specific system variables were not included in the analysed studies. Furthermore, it was not possible to identify either dynamic methods on pathways of treatment or mapping of the underlying healthcare system.

The methods most frequently used for analysis were parametric and non-parametric tests, although a general change was observed over time. It is particularly interesting to note the exceeding number of survival analysis studies conducted in the new century and the greater growth of the number of regression models used. These effects may be explained by the increasing number of patients in a study, the longer time span for follow-up data, as well as more easily used software products. It is unlikely in this case that knowledge of these methods had an effect, since Cox, for instance, had already presented his method in literature in 1972 [17]: the methods were well known during entire search timespan, which begins in 1990.

The reported median of 306 patients is sufficient for simple test statistics with regard to the effects of higher population numbers. However, only about a quarter of the studies are eligible for regression models that include several variables of interest. Yet, large sample sizes also lead to another set of problems for the interpretation for statistical analysis [18]. P-values quickly go to zero in very large samples, so that a researcher solely relying on p-values may claim support for results of no practical significance. It is likely, however, that this issue concerns only the paper by Yu [19], which considers 408,158 patients. Cox proportional regression analyses were conducted in this paper in order to compare readmission probabilities for Asians, Blacks, and Hispanics with those for Whites. The minimum follow-up time of 42 months in combination with the high number of patients is an example for good usage of Cox regression models. The low number of Poisson and Binomial Regression was also somewhat counterintuitive according to the research team’s expert opinion.

Data sources also have an impact on the method of analysis used. These sources range from, e.g., secondary hospital data to detailed interviews with patients and caregivers in combination with pathway analyses of patients. It is apparent that every healthcare/readmission modelling research question requires a prior discussion process. This is true for regression analysis, survival modelling or dynamic modelling and simulation.

The reasons for the choice of particular methods were described poorly almost throughout. One example of how such a description should be performed is provided by Barekatain et al. [20]. These authors provide a good explanation for their use of negative binomial regression: the response variable (number of readmissions) was numeric but lacked normal distribution and showed little spread. It is also very important to control the potential confounders, not least in light of the increasingly common use of big datasets with many parameters. The need to look into this aspect in the future is reflected by the fact that the International Society for Pharmacoeconomics and Outcomes Research Good Research Practices has already established a task force on these questions [21, 22]. Moreover, the use of models, which we strongly advocate, appears to be under-represented by comparison to dynamic methods in general.

Current methodology for identifying and analysing readmissions of psychiatric patients over time is largely based on classical, well known methods; it often does not take into account new technologies in data handling, statistical analysis and/or modelling and simulation. It appears that the upcoming work based on big register data, as it is defined in CEPHOS-LINK, is under-represented.

There is a range of methodological works that discuss the usability of dynamic simulation methods as well as the problems of these methods in healthcare. Kuljis et al. have provided a review of previous non-health care applications that assesses their potential usefulness to healthcare [23]. A common criticism of the way “such methods” (modelling and simulation) have been used in health care is that the approach often taken is tool-driven, starting from a “given” solution and trying to find a health care problem that fits it, instead of setting off from an existing problem and seekingan appropriate modelling and simulation solution. Barjis, who looked at healthcare simulation and its potential areas and future trends, also pointed out the problem of data collection [24]. As in statistics, a simulation model can only be as good as its input data. Data collection is the main challenge in healthcare. Yet, modelling methods open up the opportunity to integrate additional system knowledge in addition to poor data and therefore the modelling and simulation concept development makes it possible to increase knowledge via system thinking, as also discussed in [1].

The main limitation of the study was that the literature review was not designed to answer specifically methodological questions but rather those related to CEPHOS-LINK content. However, this design made it possible to conduct a detailed analysis of the current state of research and provided a reasonable basis for extending the currently used methodological pool with tools from other fields with similar questions. It is a particular strength of this review that it enables researchers on psychiatric readmission to rapidly assess which methods are available and to determine the most recent developments to increase the quality of data evaluation and therefore achieve better interpretable results.

As reported in the Methods, Ovid Medline, PsycINFO, ProQuest Health Management, OpenGrey, and Google Scholar were used for conducting the literature searches for this review. We tested extending the searches to cover also Web of Science Core Collection (ipscience.thomsonreuters.com/product/web-of-science) and Bielefeld Academic Search Engine (www.base-search.net/about/en/). Although these searches originally resulted in some additional references, after going through them carefully we found that practically none of them fit our inclusion criteria. Thus we could conclude that we had covered the area to satisfactory extent with our original searches.

Qualitative studies were excluded as this review is focused on quantitative approaches. Compared with the amount of quantitative studies on psychiatric readmission there seems to be quite a limited number of qualitative studies. They quite often use mixed methods, combining qualitative and quantitative approaches, utilizing e.g. focus groups interviews and reviewing patients’ medical histories. They also use semi-structured interviews, analysing them with a variety of techniques.

## Conclusions

The most frequently used methods for answering questions on readmission are parametric and non-parametric tests in older studies (prior to the year 2000), while regression models and survival analysis are more frequently employed in more recent papers, due among other factors to increasing study population sizes. The mathematical evaluation strategy, not the main focus on the reviewed papers, was in most cases not described satisfactorily. Many more recent methods, which are already established in other fields of health care, were missing.

The introduction of new methods to address questions regarding readmission in a psychiatric setting or even in a wider research area, like patient pathways or detailed resource planning for regionalized treatment, requires additional work on the literature in combination with data source evaluation. This must be done in interdisciplinary research groups in order to bring together the required specialists from different domains and therefore expand the current pool of methods for improving data evaluation strategies.

## Additional files

Additional file 1: Detailed search strategies: Contains the search terms for the bibliographic databases Ovid Medline, PsycINFO, ProQuest Health Management, OpenGrey (formerly SIGLE), and Google Scholar. (DOC 45 kb)
Additional file 2: Flow chart for study selection: Describes the study selection process and why records were excluded. (DOCX 35 kb)
Additional file 3: Bibliography of reviewed studies: Contains the reference list of all 407 included studies in the systematic review. (DOCX 74 kb)
Additional file 4: Additional information on cohort size and follow-up time of the reviewed studies: Contains tables with Quartiles, Minimum, Maximum and Mean of the patient sample sizes and follow-up time used in the reviewed studies. (DOCX 21 kb)
