# Supplementary material for: Statistical methods and modelling techniques for analysing hospital readmission of discharged psychiatric patients: a systematic literature review
Source: BMC Psychiatry. 2016 Nov 18;16:413. doi: 10.1186/s12888-016-1128-7 (PMC5116202; doi:10.1186/s12888-016-1128-7)
Supplement: Additional file 1: — Detailed search strategies: Contains the search terms for the bibliographic databases Ovid Medline, PsycINFO, ProQuest Health Management, OpenGrey (formerly SIGLE), and Google Scholar. (DOC 45 kb) [file 12888_2016_1128_MOESM1_ESM.doc]

**Additional file 1. Detailed search strategies**

1. **Ovid Medline**

Search date June 4 2014

PSYCHIATRIC DISORDERS

1. exp "mental disorders" (MeSH) or "mentally ill" or *stress, psychological/

2. ("mental disorder*" or "mental illness" or "mentally ill" or "mental disease" or "psychological problem*" or psychiatr* or "mental health problem*").ti.

3. 1 or 2

AND REHOSPITALISATION

4. "patient readmission" (MeSH)

5. exp "continuity of care" (MeSH)

6 ("rehospitali*" or "readmission" or "revolving door").ab

7. 4 or 5 or 6

8. 3 and 7

**Search results:**

| **#** | **Searches** | **Results** |
| --- | --- | --- |
| 1 | ("mental disorder*" or "mental illness" or "mentally ill" or "mental disease" or psychiatr*).ti. | 96314 |
| 2 | exp *"mental disorders"/ or exp *"mentally ill persons"/ | 783358 |
| 3 | 1 or 2 | 825084 |
| 4 | ("rehospitali*" or "readmission" or "repeated admission" or "revolving door").ti. | 2031 |
| 5 | *Patient Readmission/ | 3193 |
| 6 | 4 or 5 | 3756 |
| 7 | 3 and 6 | 743 |
| 8 | limit 7 to yr="1990 -Current" | 525 |
| 9 | (editorial* or letter* or news* or comment*).pt. | 1505334 |
| 10 | 8 not 9 | 490 |

# PsycINFO

# Search date June 6 2014

# PSYCHIATRIC DISORDER

# TI (mental* or "mental dis*" or "problem behav*" or "behav* problem*" or psychiatr* or psychological) OR KW (mental* or "mental dis*" or "problem behav*" or "behav* problem*" or psychiatr*)

# AND REHOSPITALISATION

# TI(rehospitali* or readmission or "continuity of care" or "revolving door") OR KW(rehospitali* or readmission or "continuity of care" or "revolving door")

# Search results:

# S4 Limiters - Publication Year: 1990-2014 Search modes - Boolean/Phrase 241

# S3 S1 OR S2 423

# S2 ( (MM "Mental Disorders" OR MM "Adjustment Disorders" OR MM "Affective Disorders" OR MM "Alexithymia" OR MM "Anxiety Disorders" OR MM "Autism" OR MM "Chronic Mental Illness" OR MM "Dementia" OR MM "Dissociative Disorders" OR MM "Eating Disorders" OR MM "Elective Mutism" OR MM "Factitious Disorders" OR MM "Gender Identity Disorder" OR MM "Hysteria" OR MM "Impulse Control Disorders" OR MM "Koro" OR MM "Mental Disorders due to General Medical Conditions" OR MM "Neurosis" OR MM "Paraphilias" OR MM "Personality Disorders" OR MM "Pervasive Developmental Disorders" OR MM "Pseudodementia" OR MM "Psychosis" OR MM "Schizoaffective Disorder") OR (MM "Psychiatric Patients") ) OR ( TI ("mental disorder*" or "mental* ill*" or "psychiatric disorder*" or "psychiatric patient*") ) 185,653

# S1 (TI(rehospitali* or readmission or "repated admission" or "repeated hospitali*" or "revolving door") OR SU("Psychiatric Hospital Readmission")) 1,263

# ProQuest Health Management

# Search date June 6 2014

PSYCHIATRIC DISORDERS

AB("mental health" or "mental dis*" or "problem behav*" or "behav* problem*" or psychiatric or psychological)

AND REHOSPITALISATION

AB("rehospitali*" or "readmission" or "continuity of care" or "revolving door")

###

# Search results:

S4 S3 limited to 1990-2014 37 references

S3 S1 AND S2 38 references

S2 (MJMESH.EXACT.EXPLODE("Patient Readmission:E.02.760.400.620") OR MJMESH.EXACT.EXPLODE("Patient Readmission:N.02.421.585.400.620")) OR ti((rehospitali* or readmission or "repated admission" or "repeated hospitali*" or "revolving door")) 882

S1 SU.EXACT("Mental disorders") OR (MJMESH.EXACT.EXPLODE("Mental Disorders Diagnosed in Childhood") OR MJMESH.EXACT.EXPLODE("Mental Disorders")) OR ti(("mental disorder*" or "mental* ill*" or "psychiatric disorder*" or "psychiatric patient*"))

# OpenGrey (formely SIGLE)

# Search date June 10 2014

PSYCHIATRIC DISORDERS

psychiatry OR "psychiatric patient" OR "mental disorder" OR "mental disease" OR "behavioral disorder"

AND REHOSPITALISATION

rehospitalisation OR readmission OR "continuity of care" OR "revolving door"

###

# Search results:

(psychiatr* OR mental*) AND (rehospitali* OR readmission OR readmitted OR “repeated admission” OR "revolving door") 3 references

1. **Google Scholar**

# Search date June 4 2014

# Search results:

limit to years 1990-

allintitle: rehospitalization psychiatric 49 references

allintitle: rehospitalization mental 21 references

allintitle: readmission mental 34 references

allintitle:readmission psychiatric 113 references

allintitle: "revolving door" mental 26 references

allintitle: "revolving door" psychiatric 14 references

allintitle: repeated admission mental 0 references

allintitle: repeated admission psychiatric 0 references
