# Supplementary material for: Statistical methods and modelling techniques for analysing hospital readmission of discharged psychiatric patients: a systematic literature review
Source: BMC Psychiatry. 2016 Nov 18;16:413. doi: 10.1186/s12888-016-1128-7 (PMC5116202; doi:10.1186/s12888-016-1128-7)
Supplement: Additional file 2: — Flow chart for study selection: Describes the study selection process and why records were excluded. (DOCX 35 kb) [file 12888_2016_1128_MOESM2_ESM.docx]

**Additional file 2. Flow chart for study selection**

Flow of studies retrieved by the systematic literature search and article selection process as performed in CEPHOS-LINK with look on used mathematical methods in psychiatric rehospitalisation evaluation:

**Identification**

Records identified through database searching
(n = 1018)

Records excluded due to lack of methodological relevance
(n = 232)

Records after duplicates removed
(n= 734)

**Screening**

Records excluded
(n = 95)

-no full text available

-not fulfilling language criteria

- discussion / commentary / opinion

- PhD thesis without methods section

Records for Methods Analysis based on title
(n = 502)

**Eligibility and Inclusion**

Studies included in qualitative synthesis
(n = 407)
