# Supplementary material for: Statistical methods and modelling techniques for analysing hospital readmission of discharged psychiatric patients: a systematic literature review
Source: BMC Psychiatry. 2016 Nov 18;16:413. doi: 10.1186/s12888-016-1128-7 (PMC5116202; doi:10.1186/s12888-016-1128-7)
Supplement: Additional file 3: — Bibliography of reviewed studies: Contains the reference list of all 407 included studies in the systematic review. (DOCX 74 kb) [file 12888_2016_1128_MOESM3_ESM.docx]

## Additional file 3. Bibliography of reviewed studies

T. Adamowski, T. Hadrys, and A. Kiejna, “[Comparison between the day-care ward and the inpatient ward in terms of treatment effectiveness based on the analysis of psychopathologic symptoms, subjective quality of life and number of rehospitalisations after discharge],” *Psychiatr.Pol.*, vol. 42, no. 4, pp. 571–581, 2008.

J. D. Adams, “Risk factors contributing to the 30 day readmission rate at the Alaska Psychiatric Institute for fiscal year 2008,” University of Alaska Anchorage, 2010.

S. J. Adams, N. L. Pitre, and R. Cieszkowski, “Who applies to regional review boards and what are the outcomes?.,” *Canadian Journal of Psychiatry - Revue Canadienne de Psychiatrie*, vol. 42, no. 1, pp. 70–76, Feb. 1997.

E. Akande, M. D. Beer, and K. Ratnajothy, “Outcome study of patients exhibiting challenging behaviours four years after discharge from a low secure mental health unit,” *Journal of Psychiatric Intensive Care*, vol. 3, no. 1, pp. 21–26, 2007.

J. S. Albrecht, J. M. Hirshon, R. Goldberg, P. Langenberg, H. R. Day, D. J. Morgan, A. C. Comer, A. D. Harris, and J. P. Furuno, “Serious mental illness and acute hospital readmission in diabetic patients,” *Am.J.Med.Qual.*, vol. 27, no. 6, pp. 503–508, 2012.

N. al-Nahedh, “Relapse among substance-abuse patients in Riyadh, Saudi Arabia.,” *Eastern Mediterranean Health Journal*, vol. 5, no. 2, pp. 241–246, 1999.

L. Appleby, P. N. Desai, D. J. Luchins, R. D. Gibbons, and D. R. Hedeker, “Length of stay and recidivism in schizophrenia: a study of public psychiatric hospital patients.,” *Am.J.Psychiatry*, vol. 150, no. 1, pp. 72–76, 1993.

C. Arfken, L. L. Zeman, L. Yeager, E. Mischel, and A. Amirsadri, “Frequent visitors to psychiatric emergency services: staff attitudes and temporal patterns.,” *J.Behav.Health Serv.Res.*, vol. 29, no. 4, pp. 490–496, 2002.

H. E. Armstrong, G. B. Cox, B. A. Short, and D. J. Allmon, “A comparative evaluation of two day treatment programs,” *Psychosocial Rehabilitation Journal*, vol. 14, no. 4, pp. 53–67, 1991.

P. M. Averill, D. R. Hopko, D. R. Small, H. B. Greenlee, and R. V. Varner, “The role of psychometric data in predicting inpatient mental health service utilization.,” *Psychiatr.Q.*, vol. 72, no. 3, pp. 215–235, 2001.

J. Baillargeon, I. Binswanger, J. Penn, B. Williams, and O. Murray, “Psychiatric disorders and repeat incarcerations: the revolving prison door,” *Am.J.Psychiatry*, vol. 166, no. 1, pp. 103–109, 2009.

M. Bandeira, “Reinserção de doentes mentais na comunidade: fatores determinantes das re-hospitalizações. = Reintegration of mentally ill patients into the community: Factors determining the probability of rehospitalization,” *J.Bras.Psiquiatr.*, vol. 42, no. 9, pp. 491–498, 1993.

S. Banks, J. Pandiani, and J. Bramley, “Approaches to risk-adjusting outcome measures applied to criminal justice involvement after 44• RISK-ADJUSTED MENTAL HEALTH OUTCOMES 435 risk-adjustment of rehospitalization rates,” *Ment.Health Serv.Res.*, vol. 3, no. Journal Article, pp. 15–24, 2001.

M. Barekatain, M. R. Maracy, R. Hassannejad, and R. Hosseini, “Factors Associated with Readmission of Patients at a University Hospital Psychiatric Ward in Iran,” *Psychiatry journal*, vol. 2013, no. Journal Article, 2013.

E. Barker, D. Robinson, and R. Brautigan, “The effect of psychiatric home nurse follow-up on readmission rates of patients with depression,” *Journal of the American Psychiatric Nurses Association*, vol. 5, no. 4, pp. 111–116, 1999.

E. Barker, “The effect of psychiatric home nurse follow-up on readmission rates of the mentally ill,” 1996.

K. Baron and J. R. Hays, “Characteristics of readmitted psychiatric inpatients.,” *Psychol.Rep.*, vol. 93, no. 1, pp. 235–238, 2003.

K. L. Baron, “Examination of sociodemographic, cognitive, and personality variables as predictors of psychiatric hospital readmissions,” ProQuest Information & Learning, US, 1998.

A. Batalla, C. Garcia-Rizo, P. Castellví, E. Fernandez-Egea, M. Yücel, E. Parellada, B. Kirkpatrick, R. Martin-Santos, and M. Bernardo, “Screening for substance use disorders in first-episode psychosis: Implications for readmission,” *Schizophr.Res.*, vol. 146, no. 1–3, pp. 125–131, 2013.

A. Bateman and P. Fonagy, “Health service utilization costs for borderline personality disorder patients treated with psychoanalytically oriented partial hospitalization versus general psychiatric care.,” *Am.J.Psychiatry*, vol. 160, no. 1, pp. 169–171, 2003.

E. A. Becker and A. Shafer, “Voluntary readmission among schizophrenic patients in the Texas state psychiatric hospital system,” *Tex.Med.*, vol. 103, no. 9, pp. 54–59, 2007.

O. Ben-Arie, A. Koch, M. Welman, and A. F. Teggin, “The effect of research on readmission to a psychiatric hospital,” *The British Journal of Psychiatry*, vol. 156, no. Journal Article, pp. 37–39, 1990.

B. B. Benda, “Factors associated with rehospitalization among veterans in a substance abuse treatment program,” *Psychiatric Services*, vol. 53, no. 9, pp. 1176–1178, 2002.

B. B. Benda, “Life-course theory of readmission of substance abusers among homeless veterans,” *Psychiatric Services*, vol. 55, no. 11, pp. 1308–1310, 2004.

J. K. Benzer, J. L. Sullivan, S. Williams, and J. F. Burgess, “One-year cost implications of using mental health care after discharge from a general medical hospitalization.,” *Psychiatric Services*, vol. 63, no. 7, pp. 672–678, 2012.

A. C. Bernardo and C. Forchuk, “Factors associated with readmission to a psychiatric facility,” *Psychiatric Services*, vol. 52, no. 8, pp. 1100–1102, 2001.

A. C. Bernet, “Predictors of psychiatric readmission among veterans at high risk of suicide: the impact of post-discharge aftercare,” *Arch.Psychiatr.Nurs.*, vol. 27, no. 5, pp. 260–261, 2013.

W. V. Bobo, C. W. Hoge, M. A. Messina, F. Pavlovcic, D. Levandowski, and T. Grieger, “Characteristics of repeat users of an inpatient psychiatry service at a large military tertiary care hospital.,” *Mil.Med.*, vol. 169, no. 8, pp. 648–653, 2004.

R. Bodén, L. Brandt, H. Kieler, M. Andersen, and J. Reutfors, “Early non-adherence to medication and other risk factors for rehospitalization in schizophrenia and schizoaffective disorder,” *Schizophr.Res.*, vol. 133, no. 1–3, pp. 36–41, 2011.

B. M. Booth, W. R. Yates, F. Petty, and K. Brown, “Patient factors predicting early alcohol-related readmissions for alcoholics: role of alcoholism severity and psychiatric co-morbidity.,” *J.Stud.Alcohol*, vol. 52, no. 1, pp. 37–43, 1991.

U. Botha, P. Oosthuizen, L. Koen, J. Joska, J. Parker, and N. Horn, “The revolving door phenomenon in psychiatry: Comparing low-frequency and high-frequency users of psychiatric inpatient services in a developing country,” no. Journal Article, 2008.

N. W. Bowersox, S. M. Saunders, and B. D. Berger, “Predictors of rehospitalization in high-utilizing patients in the VA psychiatric medical system,” *Psychiatr.Q.*, vol. 83, no. 1, pp. 53–64, 2012.

K. M. Boydell, S. A. Malcolmson, and K. Sikerbol, “Early rehospitalization,” *The Canadian Journal of Psychiatry / La Revue canadienne de psychiatrie*, vol. 36, no. 10, pp. 743–745, 1991.

P. L. Brennan, C. R. Kagay, J. J. Geppert, and R. H. Moos, “Elderly Medicare inpatients with substance use disorders characteristics and predictors of hospital readmissions over a four-year interval,” *J.Stud.Alcohol*, vol. 61, no. 6, pp. 891–895, 2000.

P. J. Brown, P. R. Recupero, and R. Stout, “PTSD substance abuse comorbidity and treatment utilization.,” *Addict.Behav.*, vol. 20, no. 2, pp. 251–254, 1995.

G. Browne, M. Courtney, and T. Meehan, “Type of housing predicts rate of readmission to hospital but not length of stay in people with schizophrenia on the Gold Coast in Queensland.,” *Australian Health Review*, vol. 27, no. 1, pp. 65–72, 2004.

R. Buffaerts, M. Sabbe, and K. Demyttenaere, “Effects of patient and health-system characteristics on community tenure of discharged psychiatric inpatients,” *Psychiatr Serv*, vol. 55, no. 6, pp. 685–690, Jun. 2004.

P. Burgess, J. Bindman, M. Leese, C. Henderson, and G. Szmukler, “Do community treatment orders for mental illness reduce readmission to hospital?: An epidemiological study,” *Soc.Psychiatry Psychiatr.Epidemiol.*, vol. 41, no. 7, pp. 574–579, 2006.

R. E. Burke, J. Donzé, and J. L. Schnipper, “Contribution of psychiatric illness and substance abuse to 30‐day readmission risk,” *Journal of Hospital Medicine*, vol. 8, no. 8, pp. 450–455, 2013.

T. Burns, J. Rugkasa, A. Molodynski, J. Dawson, K. Yeeles, M. Vazquez-Montes, M. Voysey, J. Sinclair, and S. Priebe, “Community treatment orders for patients with psychosis (OCTET): a randomised controlled trial.,” *Lancet*, vol. 381, no. 9878, pp. 1627–1633, 2013.

S. L. Byrne, G. R. Hooke, E. A. Newnham, and A. C. Page, “The effects of progress monitoring on subsequent readmission to psychiatric care: A six-month follow-up,” *J.Affect.Disord.*, vol. 137, no. 1, pp. 113–116, 2012.

W. Caan and M. Crowe, “Using readmission rates as indicators of outcome in comparing psychiatric services,” *Journal of Mental Health*, vol. 3, no. 4, pp. 521–524, 1994.

R. C. Callaghan and J. A. Cunningham, “Gender differences in detoxification: predictors of completion and re-admission.,” *J.Subst.Abuse Treat.*, vol. 23, no. 4, pp. 399–407, 2002.

R. C. Callaghan, “Risk factors associated with dropout and readmission among First Nations individuals admitted to an inpatient alcohol and drug detoxification program.,” *CMAJ Canadian Medical Association Journal*, vol. 169, no. 1, pp. 23–27, 2003.

T. Callaly, M. Hyland, T. Trauer, S. Dodd, and M. Berk, “Readmission to an acute psychiatric unit within 28 days of discharge: identifying those at risk.,” *Australian Health Review*, vol. 34, no. 3, pp. 282–285, 2010.

T. Callaly, T. Trauer, M. Hyland, T. Coombs, and M. Berk, “An examination of risk factors for readmission to acute adult mental health services within 28 days of discharge in the Australian setting,” *Australas.Psychiatry.*, vol. 19, no. 3, pp. 221–225, 2011.

E. S. Casper, J. M. Romo, and R. C. Fasnacht, “Readmission patterns of frequent users of inpatient psychiatric services,” *Psychiatric Services*, vol. 42, no. 11, pp. 1166–1167, 1991.

E. S. Casper and J. R. Regan, “Reasons for admission among six profile subgroups of recidivists of inpatient services.,” *Canadian Journal of Psychiatry - Revue Canadienne de Psychiatrie*, vol. 38, no. 10, pp. 657–661, 1993.

E. S. Casper, “Identifying multiple recidivists in a state hospital population.,” *Psychiatric Services*, vol. 46, no. 10, pp. 1074–1075, 1995.

E. Cassidy, S. Hill, and E. O’Callaghan, “Efficacy of a psychoeducational intervention in improving relatives’ knowledge about schizophrenia and reducing rehospitalisation.,” *European Psychiatry: the Journal of the Association of European Psychiatrists*, vol. 16, no. 8, pp. 446–450, 2001.

A. Castagnini, L. Foldager, and A. Bertelsen, “Long-term stability of acute and transient psychotic disorders.,” *Australian & New Zealand Journal of Psychiatry*, vol. 47, no. 1, pp. 59–64, 2013.

A. P. Castro and H. Elkis, “Rehospitalization rates of patients with schizophrenia discharged on haloperidol, risperidone or clozapine.,” *Revista Brasileira de Psiquiatria*, vol. 29, no. 3, pp. 207–212, 2007.

B. Castro, S. Bahadori, Tortelli, L. Ailam, and N. Skurnik, “Syndrome de la porte tournante en psychiatrie en 2006. = Revolving door syndrome,” *Annales Médico-Psychologiques*, vol. 165, no. 4, pp. 276–281, 2007.

M. W. C. Chandrasena R, “Discharges AMA and AWOL: A new’revolving door syndrome’,” *Psychiatric Journal of University of Ottowa*, vol. 13, no. Journal Article, pp. 154–157, 1998.

C. M. Chang, Y. Lee, Y. Lee, M. J. Yang, and J. K. Wen, “Predictors of readmission to a medical-psychiatric unit among patients with minor mental disorders,” *Chang Gung Med.J.*, vol. 24, no. 1, pp. 34–43, 2001.

J. S. Chang, K. S. Ha, K. Young Lee, Y. Sik Kim, and Y. Min Ahn, “The effects of long-term clozapine add-on therapy on the rehospitalization rate and the mood polarity patterns in bipolar disorders.,” *J.Clin.Psychiatry*, vol. 67, no. 3, pp. 461–467, 2006.

C. T. Chiang, L. F. James, and M. C. Wang, “Random weighted bootstrap method for recurrent events with informative censoring.,” *Lifetime Data Anal.*, vol. 11, no. 4, pp. 489–509, 2005.

M. J. Chinman, R. Weingarten, D. Stayner, and L. Davidson, “Chronicity reconsidered: improving person-environment fit through a consumer-run service.,” *Community Ment.Health J.*, vol. 37, no. 3, pp. 215–229, 2001.

W. H. Chou, F.-X. Yan, J. de Leon, J. Barnhill, T. Rogers, M. Cronin, M. Pho, V. Xiao, T. B. Ryder, W. W. Liu, C. Teiling, and P. J. Wedlund, “Extension of a pilot study: Impact from the cytochrome P450 2D6 polymorphism on outcome and costs associated with severe mental illness,” *J.Clin.Psychopharmacol.*, vol. 20, no. 2, pp. 246–251, 2000.

A. B. F. Christensen, “Community mental health services and sex: Utilization of psychiatric services by men and women treated in the Community Mental Health Centre in Svendborg, Funen, Denmark,” *Nordic Journal of Psychiatry*, vol. 53, no. 4, pp. 313–317, 1999.

L. A. Chwastiak, D. S. Davydow, C. L. McKibbin, E. Schur, M. Burley, M. G. McDonell, J. Roll, and K. B. Daratha, “The effect of serious mental illness on the risk of rehospitalization among patients with diabetes,” *Psychosomatics: Journal of Consultation and Liaison Psychiatry*, vol. 55, no. 2, pp. 134–143, 2014.

C. A. Claassen, T. Michael Kashner, S. K. Gilfillan, G. L. Larkin, and A. John Rush, “Psychiatric emergency service use after implementation of managed care in a public mental health system.,” *Psychiatric Services*, vol. 56, no. 6, pp. 691–698, 2005.

M. Clarke, P. Moran, F. Keogh, M. Morris, A. Kinsella, C. Larkin, D. Walsh, and E. O’Callaghan, “Seasonal influences on admissions for affective disorder and schizophrenia in Ireland: A comparison of first and readmissions,” *European Psychiatry*, vol. 14, no. 5, pp. 251–255, 1999.

K. M. Clements, J. M. Murphy, S. V. Eisen, and S.-L. T. Normand, “Comparison of self-report and clinician-rated measures of psychiatric symptoms and functioning in predicting 1-year hospital readmission,” *Administration and Policy in Mental Health and Mental Health Services Research*, vol. 33, no. 5, pp. 568–577, 2006.

C. C. Colenda, D. Trinkle, R. M. Hamer, and S. Jones, “Hospital utilization and readmission rates for geriatric and young adult patients with major depression: results from a historical cohort study.,” *Journal of Geriatric Psychiatry & Neurology*, vol. 4, no. 3, pp. 166–172, 1991.

K. C. Coley, C. S. Carter, S. V. DaPos, R. Maxwell, J. W. Wilson, and R. A. Branch, “Effectiveness of antipsychotic therapy in a naturalistic setting: A comparison between risperidone, perphenazine, and haloperidol,” *J.Clin.Psychiatry*, vol. 60, no. 12, pp. 850–856, 1999.

R. R. Conley, D. L. Kelly, R. C. Love, and R. P. McMahon, “Rehospitalization risk with second-generation and depot antipsychotics.,” *Annals of Clinical Psychiatry*, vol. 15, no. 1, pp. 23–31, 2003.

R. R. Conley, R. C. Love, D. L. Kelly, and J. J. Bartko, “Rehospitalization rates of patients recently discharged on a regimen of risperidone or clozapine.,” *Am.J.Psychiatry*, vol. 156, no. 6, pp. 863–868, 1999.

M. Cooper and S. McLees, “Can we prevent readmission? Needs for care and gaps in service provision,” *Behavioural and Cognitive Psychotherapy*, vol. 29, no. 4, pp. 497–500, 2001.

A. Cougnard, M. Parrot, S. Grolleau, E. Kalmi, A. Desage, D. Misdrahi, H. Brun-Rousseau, and H. Verdoux, “Pattern of health service utilization and predictors of readmission after a first admission for psychosis: A 2-year follow-up study,” *Acta Psychiatr.Scand.*, vol. 113, no. 4, pp. 340–349, 2006.

T. J. Craig and J. Bracken, “A case-control study of rapid readmission in a state hospital population.,” *Annals of Clinical Psychiatry*, vol. 7, no. 2, pp. 79–85, 1995.

T. J. Craig, E. J. Bromet, L. Jandorf, S. Fennig, M. Tanenberg-Karant, R. Ram, and B. Rosen, “Diagnosis, treatment, and six-month outcome status in first-admission psychosis.,” *Annals of Clinical Psychiatry*, vol. 9, no. 2, pp. 89–97, 1997.

S. M. Cummings, “Adequacy of discharge plans and rehospitalization among hospitalized dementia patients,” *Health Soc.Work*, vol. 24, no. 4, pp. 249–259, 1999.

J. L. Curtis, E. J. Millman, E. Struening, and A. D’Ercole, “Effect of case management on rehospitalization and utilization of ambulatory care services,” *Hosp.Community Psychiatry*, vol. 43, no. 9, pp. 895–899, 1992.

B. A. Daniels, K. C. Kirkby, D. A. Hay, B. J. Mowry, and I. H. Jones, “Predictability of rehospitalisation over 5 years for schizophrenia, bipolar disorder and depression.,” *Australian & New Zealand Journal of Psychiatry*, vol. 32, no. 2, pp. 281–286, 1998.

K. I. Darsow-Schutte and P. Muller, “[Number of hospitalizations according to German ‘PsychKG’ legislation has doubled in 10 years],” *Psychiatr.Prax.*, vol. 28, no. 5, pp. 226–229, 2001.

L. Davidson, D. A. Stayner, M. J. Chinman, S. Lambert, and W. H. Sledge, “Preventing relapse and readmission in psychosis: Using parients’ subjective experience in designing clinical interventions,” in *Psychosis: Psychological approaches and their effectiveness.*, Book, Section vols., B. Martindale, A. Bateman, and F. Margison, Eds. London, England: Gaskell/Royal College of Psychiatrists, 2000, pp. 134–156.

S. Davies, M. Clarke, C. Hollin, and C. Duggan, “Long-term outcomes after discharge from medium secure care: A cause for concern,” *The British Journal of Psychiatry*, vol. 191, no. Journal Article, pp. 70–74, 2007.

D. Dayson, “The TAPS project: XII. Crime, vagrancy, death and readmission of the long-term mentally ill during their first year of local reprovision,” *The British Journal of Psychiatry*, vol. 162, no. Journal Article, pp. 40–44, 1993.

K. Degen, N. Cole, L. Tamayo, and G. Dzerovych, “Intensive case management for the seriously mentally ill,” *Adm.Policy Ment.Health*, vol. 17, no. 4, pp. 265–269, 1990.

N. Dharwadkar, “Effectiveness of an assertive outreach community treatment program,” *Aust.N.Z.J.Psychiatry*, vol. 28, no. 2, pp. 244–249, 1994.

M. K. Distefano, M. W. Pryer, and J. L. Garrison, “Validity of psychiatric patients’ self-reports of rehospitalization,” *Hosp.Community Psychiatry*, vol. 42, no. 8, pp. 849–850, 1991.

M. Dixon, E. Robertson, M. George, and F. Oyebode, “Risk factors for acute psychiatric readmission,” *Psychiatric Bulletin*, vol. 21, no. 10, pp. 600–603, 1997.

S. Doering, E. Müller, W. Köpcke, A. Pietzcker, W. Gaebel, M. Linden, P. Müller, F. Müller-Spahn, J. Tegeler, and G. Schüssler, “Predictors of relapse and rehospitalization in schizophrenia and schizoaffective disorder,” *Schizophr.Bull.*, vol. 24, no. 1, pp. 87–98, 1998.

D. C. Donat and J. Haverkamp, “Treatment of psychiatric impairment complicated by co-occurring substance use: Impact on rehospitalization,” *Psychiatr.Rehabil.J.*, vol. 28, no. 1, pp. 78–82, 2004.

D. C. Donat, “Personality traits and psychiatric rehospitalization: A two-year follow-up,” *J.Pers.Assess.*, vol. 68, no. 3, pp. 703–711, 1997.

A. Downing and B. Hatfield, “The Care Programme approach: Dimensions of evaluation,” *British Journal of Social Work*, vol. 29, no. 6, pp. 841–860, 1999.

P. W. Durance, T. B. Gibson, M. L. Davis-Sacks, and R. K. Homan, “Multifacility utilization by the chronically mentally ill in the Department of Veterans Affairs,” *J.Ment.Health Adm.*, vol. 19, no. 2, pp. 178–194, 1992.

W. W. Eaton, P. B. Mortensen, H. Herrman, H. Freeman, W. Bilker, P. Burgess, and K. Wooff, “Long-term course of hospitalization for schizophrenia: Part I. Risk for rehospitalization.,” *Schizophr.Bull.*, vol. 18, no. 2, pp. 217–228, 1992.

B. Eikelmann and T. Reker, “A modern therapeutic approach for chronically mentally ill patients: Results of a four-year prospective study,” *Acta Psychiatr.Scand.*, vol. 84, no. 4, pp. 357–363, 1991.

E. T. Emmer and R. P. Jemelka, “A proportional hazards model for the prediction of psychiatric rehospitalization,” no. Journal Article, 2003.

A. Espadas, “An Investigation of the Unique Demographic and Clinical Characteristic of a Mental Health Population with Multiple Psychiatric Hospital Admission: A Study of Rehospitalization Rates,” 2006.

S. M. Essock, W. A. Hargreaves, F.-A. Dohm, J. Goethe, L. Carver, and L. Hipshman, “Clozapine eligibility among state hospital patients,” *Schizophr.Bull.*, vol. 22, no. 1, pp. 15–25, 1996.

W. K. Fakhoury, I. White, S. Priebe, and G. PLAO Study, “Be good to your patient: how the therapeutic relationship in the treatment of patients admitted to assertive outreach affects rehospitalization.,” *Journal of Nervous & Mental Disease*, vol. 195, no. 9, pp. 789–791, 2007.

S. Feigon and J. R. Hays, “Prediction of readmission of psychiatric inpatients,” *Psychol.Rep.*, vol. 93, no. 3, pp. 816–818, 2003.

S. Fennig, J. Rabinowitz, and S. Fennig, “Involuntary first admission of patients with schizophrenia as a predictor of future admissions.,” *Psychiatric Services*, vol. 50, no. 8, pp. 1049–1052, 1999.

S. Fennig, T. J. Craig, M. Tanenberg-Karant, L. Jandorf, B. Rosen, and E. Bromet, “Medication treatment in first-admission patients with psychotic affective disorders: Preliminary findings on research-facility diagnostic agreement and rehospitalization,” *Annals of Clinical Psychiatry*, vol. 7, no. 2, pp. 87–90, 1995.

G. A. Fernandez and S. Nygard, “Impact of involuntary outpatient commitment on the revolving-door syndrome in North Carolina.,” *Hosp.Community Psychiatry*, vol. 41, no. 9, pp. 1001–1004, 1990.

R. Figueroa, J. Harman, and J. Engberg, “Use of Claims Data to Examine the Impact of Length of Inpatient Psychiatric Stay on Readmission Rate,” *Psychiatric Services*, vol. 55, no. 5, pp. 560–565, 2004.

W. H. Fisher, J. L. Geller, F. Altaffer, and M. B. Bennett, “The relationship between community resources and state hospital recidivism.,” *Am.J.Psychiatry*, vol. 149, no. 3, pp. 385–390, 1992.

D. Frank, J. C. Perry, D. Kean, M. Sigman, and K. Geagea, “Effects of Compulsory Treatment Orders on Time to Hospital Readmission,” *Psychiatric Services*, vol. 56, no. 7, pp. 867–869, 2005.

N. Frasure-Smith, F. Lesperance, G. Gravel, A. Masson, M. Juneau, M. Talajic, and M. G. Bourassa, “Depression and health-care costs during the first year following myocardial infarction.,” *J.Psychosom.Res.*, vol. 48, no. 4–5, pp. 471–478, 2000.

R. S. Frazier and E. S. Casper, “Best Practices: A Comparative Study of Clinical Events as Triggers for Psychiatric Readmission of Multiple Recidivists,” *Psychiatric Services*, vol. 49, no. 11, pp. 1423–1425, 1998.

S. Frederick, K. Caldwell, and D. M. Rubio, “Home-based treatment, rates of ambulatory follow-up, and psychiatric rehospitalization in a Medicaid managed care population,” *The journal of behavioral health services & research*, vol. 29, no. 4, pp. 466–475, 2002.

J. J. Fu, M. Herme, J. A. Wickersham, A. Zelenev, A. Althoff, N. D. Zaller, A. R. Bazazi, A. K. Avery, J. Porterfield, A. O. Jordan, D. Simon-Levine, M. Lyman, and F. L. Altice, “Understanding the revolving door: individual and structural-level predictors of recidivism among individuals with HIV leaving jail.,” *AIDS & Behavior*, vol. 17, no. Suppl 2, pp. S145–55, 2013.

R. L. Fuller, G. Atkinson, E. C. McCullough, and J. S. Hughes, “Hospital readmission rates: the impacts of age, payer, and mental health diagnoses,” *J.Ambul.Care.Manage.*, vol. 36, no. 2, pp. 147–155, 2013.

F. L. Gastal, S. B. Andreoli, M. I. Quintana, M. Almeida Gameiro, S. O. Leite, and J. McGrath, “Predicting the revolving door phenomenon among patients with schizophrenic, affective disorders and non-organic psychoses.,” *Rev.Saude Publica*, vol. 34, no. 3, pp. 280–285, 2000.

C. A. Gbiri, F. A. Badru, H. T. Ladapo, and A. A. Gbiri, “Socio-economic correlates of relapsed patients admitted in a Nigerian mental health institution.,” *Int.J.Psychiatry Clin.Pract.*, vol. 15, no. 1, pp. 19–26, 2011.

J. L. Geller, “A report on the ‘worst’ state hospital recidivists in the U.S,” *Hosp.Community Psychiatry*, vol. 43, no. 9, pp. 904–908, 1992.

J. L. Geller, W. H. Fisher, M. McDermeit, and J. M. Brown, “The effects of public managed care on patterns of intensive use of inpatient psychiatric services.,” *Psychiatric Services*, vol. 49, no. 3, pp. 327–332, 1998.

L. S. Gillis, A. Koch, and M. Joyi, “The value and cost-effectiveness of a home-visiting programme for psychiatric patients.,” *South African Medical Journal*, vol. 77, no. 6, pp. 309–310, 1990.

W. M. Glazer and L. Ereshefsky, “A pharmacoeconomic model of outpatient antipsychotic therapy in ‘revolving door’ schizophrenic patients.,” *J.Clin.Psychiatry*, vol. 57, no. 8, pp. 337–345, 1996.

J. W. Goethe, E. A. Dornelas, and E. H. Fischer, “A cluster analytic study of functional outcome after psychiatric hospitalization,” *Compr.Psychiatry*, vol. 37, no. 2, pp. 115–121, 1996.

R. Goldbeck, M. Asif, M. Sanderson, and C. Farquharson, “Alcohol and drug misuse, risk of re-admission to a general hospital and psychiatric contact.,” *Scott.Med.J.*, vol. 57, no. 1, p. 60, 2012.

C. Gooch and J. Leff, “Factors affecting the success of community placement: the TAPS project 26.,” *Psychol.Med.*, vol. 26, no. 3, pp. 511–520, 1996.

W. A. Goodpastor and B. K. Hare, “Factors associated with multiple readmissions to an urban public psychiatric hospital,” *Hosp.Community Psychiatry*, vol. 42, no. 1, pp. 85–87, 1991.

J. Graca, C. Klut, B. Trancas, N. Borja-Santos, and G. Cardoso, “Characteristics of frequent users of an acute psychiatric inpatient unit: a five-year study in Portugal.,” *Psychiatric Services*, vol. 64, no. 2, pp. 192–195, 2013.

R. F. Grace, G. Shenfield, and C. Tennant, “Cannabis and psychosis in acute psychiatric admissions,” *Drug Alcohol Rev.*, vol. 19, no. 3, pp. 287–290, 2000.

A. Grinshpoon, M. Z. Abramowitz, Y. Lerner, and N. Zilber, “Re-hospitalization of first-in-life admitted schizophrenic patients before and after rehabilitation legislation: a comparison of two national cohorts.,” *Social Psychiatry & Psychiatric Epidemiology*, vol. 42, no. 5, pp. 355–359, 2007.

A. Grinshpoon, Y. Shershevsky, D. Levinson, and A. Ponizovsky, “Should Patients with Chronic Psychiatric Disorders Remain in Hospital? Results From a Service Inquiry,” *Isr.J.Psychiatry Relat.Sci.*, vol. 40, no. 4, pp. 268–273, 2003.

N. C. Guan and M. S. B. Yusoff, “Evaluating the Added Value of a Predictor in Psychiatric Early Readmission Rate Using Areas under the Receiver Operating Characteristic Curves and Net Reclassification Improvement.,” *Int.Med.J.*, vol. 19, no. 2, 2012.

D. Gunnell, K. Hawton, D. Ho, J. Evans, S. O’Connor, J. Potokar, J. Donovan, and N. Kapur, “Hospital admissions for self harm after discharge from psychiatric inpatient care: cohort study.,” *BMJ*, vol. 337, no. Journal Article, p. a2278, 2008.

S. Gutwinski, P. Muller, and M. Koller, “[Intervals between hospitalisations in schizophrenia patients under antipsychotics in depot-form versus oral second generation antipsychotics],” *Psychiatr.Prax.*, vol. 34, no. 6, pp. 289–291, 2007.

T. L. Hafemeister and S. M. Banks, “Methodological advances in the use of recidivism rates to assess mental health treatment programs.,” *J.Ment.Health Adm.*, vol. 23, no. 2, pp. 190–206, 1996.

R. J. Hafner and G. Holme, “The influence of a therapeutic community on psychiatric disorder,” *J.Clin.Psychol.*, vol. 52, no. 4, pp. 461–468, 1996.

A. Hamden, R. Newton, K. McCauley-Elsom, and W. Cross, “Is deinstitutionalization working in our community?,” *International Journal of Mental Health Nursing*, vol. 20, no. 4, pp. 274–283, 2011.

P. Harrison-Read, B. Lucas, P. Tyrer, J. Ray, K. Shipley, S. Simmonds, M. Knapp, A. Lowin, A. Patel, and M. Hickman, “Heavy users of acute psychiatric beds: randomized controlled trial of enhanced community management in an outer London borough.,” *Psychol.Med.*, vol. 32, no. 3, pp. 403–416, 2002.

M. Hassan and M. J. Lage, “Risk of rehospitalization among bipolar disorder patients who are nonadherent to antipsychotic therapy after hospital discharge.,” *American Journal of Health-System Pharmacy*, vol. 66, no. 4, pp. 358–365, 2009.

T. W. Haywood, H. M. Kravitz, L. S. Grossman, J. L. Cavanaugh, J. M. Davis, and D. A. Lewis, “Predicting the ‘revolving door’ phenomenon among patients with schizophrenic, schizoaffective, and affective disorders,” *Am.J.Psychiatry*, vol. 152, no. 6, pp. 856–861, 1995.

O. Heeren, L. Dixon, S. Gavirneni, and W. T. Regenold, “The association between decreasing length of stay and readmission rate on a psychogeriatric unit,” *Psychiatric Services*, vol. 53, no. 1, pp. 76–79, 2002.

T. Heggestad, S. E. Lilleeng, and T. Ruud, “Patterns of mental health care utilisation: distribution of services and its predictability from routine data.,” *Social Psychiatry & Psychiatric Epidemiology*, vol. 46, no. 12, pp. 1275–1282, 2011.

T. Heggestad, “Operating conditions of psychiatric hospitals and early readmission--effects of high patient turnover,” *Acta Psychiatr.Scand.*, vol. 103, no. 3, pp. 196–202, 2001.

M. S. Hendryx, R. Moore, T. Leeper, M. Reynolds, and S. Davis, “An examination of methods for risk-adjustment of rehospitalization rates.,” *Ment.Health Serv.Res.*, vol. 3, no. 1, pp. 15–24, 2001.

M. S. Hendryx, J. E. Russo, B. Stegner, D. G. Dyck, R. K. Ries, and P. Roy-Byrne, “Predicting rehospitalization and outpatient services from administration and clinical databases.,” *J.Behav.Health Serv.Res.*, vol. 30, no. 3, pp. 342–351, 2003.

M. Herceg, V. Jukic, D. Vidovic, V. Erdeljic, I. Celic, O. Kozumplik, D. Bagaric, and M. Silobrcic Radic, “Two-year rehospitalization rates of patients with newly diagnosed or chronic schizophrenia on atypical or typical antipsychotic drugs: retrospective cohort study.,” *Croat.Med.J.*, vol. 49, no. 2, pp. 215–223, 2008.

V. A. Hiday and T. Scheid-Cook, “Outpatient commitment for ‘revolving door’ patients: Compliance and treatment,” *J.Nerv.Ment.Dis.*, vol. 179, no. 2, pp. 83–88, 1991.

R. Hodgson, M. Lewis, and A. Boardman, “Prediction of readmission to acute psychiatric units,” *Soc.Psychiatry Psychiatr.Epidemiol.*, vol. 36, no. 6, pp. 304–309, 2001.

M. Hofecker-Fallahpour, A. Eichenberger, and W. Rössler, “Die nachtklinik—ein Dinosaurier der Reformpsychiatrie? = Aspects of vocational rehabilitation in a night partial hospitalisation programme,” *Schweizer Archiv für Neurologie und Psychiatrie*, vol. 152, no. 1, pp. 5–10, 2001.

H. Hoffmann, “Age and other factors relevant to the rehospitalization of schizophrenic outpatients.,” *Acta Psychiatr.Scand.*, vol. 89, no. 3, pp. 205–210, 1994.

P. Holmes-Eber and S. Riger, “Hospitalization and the composition of mental patients’ social networks.,” *Schizophr.Bull.*, vol. 16, no. 1, pp. 157–164, 1990.

W. P. Hornung, U. Franzen, R. Lemke, C. Wiesemann, and G. Buchkremer, “[Can psycho-education of chronic schizophrenic patients have a short-term effect on drug-related attitude and behavior?],” *Psychiatr.Prax.*, vol. 20, no. 4, pp. 152–154, 1993.

K. Humphreys and K. R. Weingardt, “Assessing readmission to substance abuse treatment as an indicator of outcome and program performance.,” *Psychiatric Services*, vol. 51, no. 12, pp. 1568–1569, 2000.

J. Husted and S. Wentler, “Changing self-perception: success in independent living for individuals with chronic mental illness.,” *Psychol.Rep.*, vol. 99, no. 2, pp. 562–568, 2006.

J. Husted, S. Wentler, G. Allen, and D. Longhenry, “The effectiveness of community support programs in rural Minnesota: A ten year longitudinal study,” *Psychiatr.Rehabil.J.*, vol. 24, no. 1, pp. 69–72, 2000.

M. Ilgen, K. Hu, R. Moos, and J. McKellar, “Continuing care utilization and readmission to inpatient psychiatric treatment in patients with co-occurring psychiatric and substance use disorders,” *Psychiatric Services*, vol. 59, no. Journal Article, pp. 982–988, 2008.

C. Irmiter, K. L. Barry, K. Cohen, and F. C. Blow, “Sixteen-year predictors of substance use disorder diagnoses for patients with mental health disorders.,” *Substance Abuse*, vol. 30, no. 1, pp. 40–46, 2009.

C. Irmiter, J. F. McCarthy, K. L. Barry, S. Soliman, and F. C. Blow, “Reinstitutionalization following psychiatric discharge among VA patients with serious mental illness: a national longitudinal study.,” *Psychiatr.Q.*, vol. 78, no. 4, pp. 279–286, 2007.

H. Ito, S. V. Eisen, and L. I. Sederer, “Acute care psychiatry at McLean hospital, Massachusetts,” *Int.Med.J.*, vol. 8, no. 2, pp. 91–96, 2001.

C. T. Jackson, D. Fein, S. M. Essock, and K. T. Mueser, “The effects of cognitive impairment and substance abuse on psychiatric hospitalizations.,” *Community Ment.Health J.*, vol. 37, no. 4, pp. 303–312, 2001.

H. Jackson, P. McGorry, J. Edwards, C. Hulbert, L. Henry, S. Harrigan, P. Dudgeon, S. Francey, D. Maude, J. Cocks, E. Killackey, and P. Power, “A controlled trial of cognitively oriented psychotherapy for early psychosis (COPE) with four-year follow-up readmission data,” *Psychol.Med.*, vol. 35, no. 9, pp. 1295–1306, 2005.

W. Jiang, J. Alexander, E. Christopher, M. Kuchibhatla, L. H. Gaulden, M. S. Cuffe, M. A. Blazing, C. Davenport, R. M. Califf, R. R. Krishnan, and C. M. O’Connor, “Relationship of depression to increased risk of mortality and rehospitalization in patients with congestive heart failure.,” *Arch.Intern.Med.*, vol. 161, no. 15, pp. 1849–1856, 2001.

R. Jones, W. R. Yates, and M. Zhou, “Readmission rates for adjustment disorders: Comparison with other mood disorders,” *J.Affect.Disord.*, vol. 71, no. 1–3, pp. 199–203, 2002.

A. Juven-Wetzler, D. Bar-Ziv, S. Cwikel-Hamzany, A. Abudy, N. Peri, and J. Zohar, “A pilot study of the ‘Continuation of Care’ model in ‘revolving-door’ patients,” *European Psychiatry*, vol. 27, no. 4, pp. 229–233, 2012.

W. Kaiser, K. Hoffmann, M. Isermann, and S. Priebe, “[Long-term patients in supported housing after deinstitutionalisation--part V of the Berlin Deinstitutionalisation Study],” *Psychiatr.Prax.*, vol. 28, no. 5, pp. 235–243, 2001.

M. Kastrup, “The use of a psychiatric register in predicting the outcome" revolving door patients,” *Acta Psychiatr.Scand.*, vol. 76, no. Journal Article, pp. 552–560, 1995.

S. Kent and P. Yellowlees, “Psychiatric and social reasons for frequent rehospitalization,” *Hosp.Community Psychiatry*, vol. 45, no. 4, pp. 347–350, 1994.

S. G. Kertesz, N. J. Horton, P. D. Friedmann, R. Saitz, and J. H. Samet, “Slowing the revolving door: stabilization programs reduce homeless persons’ substance use after detoxification.,” *J.Subst.Abuse Treat.*, vol. 24, no. 3, pp. 197–207, 2003.

L. V. Kessing, E. W. Olsen, P. B. Mortensen, and P. K. Andersen, “Dementia in affective disorder: A case-register study,” *Acta Psychiatr.Scand.*, vol. 100, no. 3, pp. 176–185, 1999.

L. V. Kessing, E. W. Olsen, and P. K. Andersen, “Recurrence in affective disorder: analyses with frailty models.,” *Am.J.Epidemiol.*, vol. 149, no. 5, pp. 404–411, 1999.

H. Kikuchi, M. Abo, E. Kumakura, N. Kubota, and M. Nagano, “Efficacy of continuous follow-up for preventing the involuntary readmission of psychiatric patients in japan: A retrospective cohort study,” *Int.J.Soc.Psychiatry*, vol. 59, no. 3, pp. 288–295, 2013.

R. Kilian and M. C. Angermeyer, “[The impact of antipsychotic medication on the incidence and the costs of inpatient treatment in people with schizophrenia: results from a prospective observational study],” *Psychiatr.Prax.*, vol. 31, no. 3, pp. 138–146, 2004.

R. Kilian, C. Roick, and M. C. Angermeyer, “[The impact of the study design and the sampling procedure on the assessment of mental health services],” *Nervenarzt*, vol. 74, no. 7, pp. 561–570, 2003.

J. H. Kim, D. Kim, and S. R. Marder, “Time to rehospitalization of clozapine versus risperidone in the naturalistic treatment of comorbid alcohol use disorder and schizophrenia.,” *Prog.Neuropsychopharmacol.Biol.Psychiatry*, vol. 32, no. 4, pp. 984–988, 2008.

D. R. Kivlahan, J. R. Heiman, R. C. Wright, J. W. Mundt, and J. A. Shupe, “Treatment cost and rehospitalization rate in schizophrenic outpatients with a history of substance abuse.,” *Hosp.Community Psychiatry*, vol. 42, no. 6, pp. 609–614, 1991.

M. Kobayashi, H. Ito, Y. Okumura, K. Mayahara, Y. Matsumoto, and J. Hirakawa, “Hospital readmission in first-time admitted patients with schizophrenia: Smoking patients had higher hospital readmission rate than non-smoking patients,” *Int.J.Psychiatry Med.*, vol. 40, no. 3, pp. 247–257, 2010.

A. Kolbasovsky, “Reducing 30-day inpatient psychiatric recidivism and associated costs through intensive case management.,” *Professional Case Management*, vol. 14, no. 2, pp. 96–105, 2009.

H. Komatsu, Y. Sekine, N. Okamura, N. Kanahara, K. Okita, S. Matsubara, T. Hirata, T. Komiyama, H. Watanabe, Y. Minabe, and M. Iyo, “Effectiveness of Information Technology Aided Relapse Prevention Programme in Schizophrenia excluding the effect of user adherence: a randomized controlled trial.,” *Schizophr.Res.*, vol. 150, no. 1, pp. 240–244, 2013.

P. König, C. Geiger, A. Künz, E. Künzle, I. Ludescher, S. Moosbrugger, V. Popadic, A. Reinthaler, G. Senft, and E. Swoboda, “Demografische und klinische Charakteristika wieder aufgenommener psychisch Kranker 1 Teil: Demografische Variablen. = Demographic and Clinical Characteristics of Readmitted Psychiatric Inpatients: Demographic Variables,” *Krankenhauspsychiatrie*, vol. 14, no. 4, pp. 143–148, 2003.

J. A. Korkeila, V. Lehtinen, T. Tuori, and H. Helenius, “Frequently hospitalised psychiatric patients: A study of predictive factors,” *Soc.Psychiatry Psychiatr.Epidemiol.*, vol. 33, no. 11, pp. 528–534, 1998.

J. A. Korkeila, H. Karlsson, and H. Kujari, “Factors predicting readmissions in personality disorders and other nonpsychotic illness: A retrospective study on 64 first-ever admissions to the Psychiatric Clinic of Turku, Finland,” *Acta Psychiatr.Scand.*, vol. 92, no. 2, pp. 138–144, 1995.

P. Kottsieper, “Predicting initial aftercare appointment adherence and rehospitalization for individuals with serious mental illness discharged from an acute inpatient stay,” ProQuest Information & Learning, US, 2006.

M. Krautgartner, M. Scherer, and H. Katschnig, “[Days in psychiatric hospitals: who consumes most of them? A five-year record linkage study of ‘Heavy Users’ in an Austrian province],” *Psychiatr.Prax.*, vol. 29, no. 7, pp. 355–363, 2002.

T. J. Kreys, T. J. Fabian, M. I. Saul, R. Haskett, and K. C. Coley, “An evaluation of inpatient treatment continuation and hospital readmission rates in patients with bipolar disorder treated with aripiprazole or quetiapine.,” *Journal of Psychiatric Practice*, vol. 19, no. 4, pp. 288–295, 2013.

H. L. Krober, R. Adam, and R. Scheidt, “[Risk factors for recurrence in bipolar manic-depressive patients],” *Nervenarzt*, vol. 69, no. 1, pp. 46–52, 1998.

S. Kumar, E. Robinson, and V. Kumar Sinha, “What leads to frequent re-hospitalisation when community care is not well developed?.,” *Social Psychiatry & Psychiatric Epidemiology*, vol. 37, no. 9, pp. 435–440, 2002.

E. Kuno, A. B. Rothbard, and R. G. Sands, “Service components of case management which reduce inpatient care use for persons with serious mental illness,” *Community Ment.Health J.*, vol. 35, no. 2, pp. 153–167, 1999.

L. A. Labbate and M. E. Doyle, “Recidivism in major depressive disorder,” *Psychotherapy & Psychosomatics*, vol. 66, no. 3, pp. 145–149, 1997.

M. H. Lafeuille, F. Laliberte-Auger, P. Lefebvre, C. Frois, J. Fastenau, and M. S. Duh, “Impact of atypical long-acting injectable versus oral antipsychotics on rehospitalization rates and emergency room visits among relapsed schizophrenia patients: a retrospective database analysis,” *BMC Psychiatry*, vol. 13, no. Journal Article, p. 221, 2013.

T. P. Lang, J. E. Rohrer, and P. A. Rioux, “Multifaceted inpatient psychiatry approach to reducing readmissions: a pilot study.,” *Journal of Rural Health*, vol. 25, no. 3, pp. 309–313, 2009.

B. Lay, C. Lauber, and W. Rössler, “Prediction of in-patient use in first-admitted patients with psychosis,” *European Psychiatry*, vol. 21, no. 6, pp. 401–409, 2006.

A. S. Lee, C. Duggan, and R. M. Murray, “Can one predict the long-term outcome of hospitalized depressives?,” *Journal of Psychopharmacology*, vol. 6, no. 2, pp. 300–303, 1992.

H. C. Lee and H. C. Lin, “Is the volume-outcome relationship sustained in psychiatric care?,” *Social Psychiatry & Psychiatric Epidemiology*, vol. 42, no. 8, pp. 669–672, 2007.

Y. Lerner and N. Zilber, “Predictors of cumulative length of psychiatric inpatient stay over one year: a national case register study,” *Israel Journal of Psychiatry & Related Sciences*, vol. 47, no. 4, pp. 304–307, 2010.

J. L. Levenson, R. M. Hamer, and L. F. Rossiter, “Psychopathology and pain in medical in-patients predict resource use during hospitalization but not rehospitalization,” *J.Psychosom.Res.*, vol. 36, no. 6, pp. 585–592, 1992.

T. Lewis and P. R. Joyce, “The new revolving-door patients: Results from a national cohort of first admissions,” *Acta Psychiatr.Scand.*, vol. 82, no. 2, pp. 130–135, 1990.

X. Li, H. Sun, D. C. Marsh, and A. H. Anis, “Factors associated with seeking readmission among clients admitted to medical withdrawal management.,” *Substance Abuse*, vol. 29, no. 4, pp. 65–72, 2008.

P. Lichtenberg, D. Levinson, Y. Sharshevsky, D. Feldman, and M. Lachman, “Clinical case management of revolving door patients - a semi-randomized study.,” *Acta Psychiatr.Scand.*, vol. 117, no. 6, pp. 449–454, 2008.

C. H. Lin, C. C. Chen, S. Y. Wang, S. C. Lin, M. C. Chen, and C. H. Lin, “Factors affecting time to rehospitalization in Han Chinese patients with schizophrenic disorder in Taiwan.,” *Kaohsiung J.Med.Sci.*, vol. 24, no. 8, pp. 408–414, 2008.

C. H. Lin, W. L. Chen, C. M. Lin, M. D. Lee, M. C. Ko, and C. Y. Li, “Predictors of psychiatric readmissions in the short- and long-term: a population-based study in Taiwan.,” *Clinics (Sao Paulo, Brazil)*, vol. 65, no. 5, pp. 481–489, 2010.

C. H. Lin, Y. S. Chen, C. H. Lin, and K. S. Lin, “Factors affecting time to rehospitalization for patients with major depressive disorder.,” *Psychiatry & Clinical Neurosciences*, vol. 61, no. 3, pp. 249–254, 2007.

C. H. Lin, C. J. Huang, Y. H. Huang, and C. C. Chen, “Time to rehospitalization of schizophrenia patients with alcohol use disorders.,” *Acta Psychiatr.Scand.*, vol. 128, no. 1, pp. 94–95, 2013.

C. H. Lin, K. S. Lin, C. Y. Lin, M. C. Chen, and H. Y. Lane, “Time to rehospitalization in patients with major depressive disorder taking venlafaxine or fluoxetine.,” *J.Clin.Psychiatry*, vol. 69, no. 1, pp. 54–59, 2008.

C. H. Lin, S. C. Lin, M. C. Chen, and S. Y. Wang, “Comparison of time to rehospitalization among schizophrenic patients discharged on typical antipsychotics, clozapine or risperidone.,” *Journal of the Chinese Medical Association: JCMA*, vol. 69, no. 6, pp. 264–269, 2006.

C.-H. Lin, M.-C. Chen, L.-S. Chou, C.-H. Lin, C.-C. Chen, and H.-Y. Lane, “Time to rehospitalization in patients with major depression vs. those with schizophrenia or bipolar I disorder in a public psychiatric hospital,” *Psychiatry Res.*, vol. 180, no. 2, pp. 74–79, 2010.

C.-H. Lin, C.-C. Kuo, R.-Y. Liu, C.-W. Huang, and C.-C. Chen, “Factors affecting time to rehospitalization for Chinese patients with bipolar I disorder in Taiwan,” *Aust.N.Z.J.Psychiatry*, vol. 43, no. 10, pp. 927–933, 2009.

H. C. Lin and H. C. Lee, “The association between timely outpatient visits and the likelihood of rehospitalization for schizophrenia patients.,” *Am.J.Orthopsychiatry*, vol. 78, no. 4, pp. 494–497, 2008.

H. C. Lin, W. H. Tian, C. S. Chen, T. C. Liu, S. Y. Tsai, and H. C. Lee, “The association between readmission rates and length of stay for schizophrenia: a 3-year population-based study.,” *Schizophr.Res.*, vol. 83, no. 2–3, pp. 211–214, 2006.

H.-C. Lin and H.-C. Lee, “Psychiatrists’ caseload volume, length of stay and mental healthcare readmission rates: A three-year population-based study,” *Psychiatry Res.*, vol. 166, no. 1, pp. 15–23, 2009.

J. D. Little, J. Munday, M. R. Atkins, and A. Khalid, “Does electrode placement predict time to rehospitalization?.,” *J.ECT*, vol. 20, no. 4, pp. 213–218, 2004.

S. Li-Yu, D. E. Biegel, and J. A. Johnsen, “Predictors of psychiatric rehospitalization for persons with serious and persistent mental illness,” *Psychiatr.Rehabil.J.*, vol. 22, no. 2, pp. 155–166, 1998.

A. A. Loch, “Stigma and higher rates of psychiatric re-hospitalization: São Paulo public mental health system,” *Revista Brasileira de Psiquiatria*, vol. 34, no. 2, pp. 185–192, 2012.

B. Luchansky, L. He, A. Krupski, and K. D. Stark, “Predicting readmission to substance abuse treatment using state information systems. The impact of client and treatment characteristics.,” *J.Subst.Abuse*, vol. 12, no. 3, pp. 255–270, 2000.

B. Luchansky, L. He, D. Longhi, A. Krupski, and K. D. Stark, “Treatment readmissions and criminal recidivism in youth following participation in chemical dependency treatment.,” *Journal of Addictive Diseases*, vol. 25, no. 1, pp. 87–94, 2006.

P. H. Lysaker, M. D. Bell, S. Bioty, and W. S. Zito, “Performance on the Wisconsin Card Sorting Test as a predictor of rehospitalization in schizophrenia,” *J.Nerv.Ment.Dis.*, vol. 184, no. 5, pp. 319–321, 1996.

E. Madlung, C. Haring, J. A. Crespo, A. Saria, P. Grubinger, and G. Zernig, “Methadone doses upon multiple readmissions to inpatient detoxification: Clinical evidence for very moderate opioid tolerance.,” *Pharmacology*, vol. 78, no. 1, pp. 38–43, 2006.

R. Mahendran, Mythily, S.-A. Chong, and Y. H. Chan, “Brief Communication: Factors Affecting Rehospitalisation in Psychiatric Patients in Singapore,” *Int.J.Soc.Psychiatry*, vol. 51, no. 2, pp. 101–105, 2005.

A. Mares and J. McGuire, “Reducing psychiatric hospitalization among mentally ill veterans living in board-and-care homes,” *Psychiatric Services*, vol. 51, no. 7, pp. 914–921, 2000.

S. Marom, H. Munitz, P. B. Jones, A. Weizman, and H. Hermesh, “Expressed emotion: relevance to rehospitalization in schizophrenia over 7 years.,” *Schizophr.Bull.*, vol. 31, no. 3, pp. 751–758, 2005.

J. M. Martinez-Ortega, L. Gutierrez-Rojas, D. Jurado, A. Higueras, F. J. Diaz, and M. Gurpegui, “Factors associated with frequent psychiatric admissions in a general hospital in Spain.,” *Int.J.Soc.Psychiatry*, vol. 58, no. 5, pp. 532–535, 2012.

F. Martín Ortíz, C. Duarte Diéguez, and F. Rius Díaz, “Tiempo de ingreso y readmisiones en pacientes psiquiátricos: un estudio sobre grupos diagnósticos. = Length of study and readmissions of psychiatric patients: A study of the diagnostic groups,” *Anales de Psiquiatría*, vol. 11, no. 4, pp. 140–144, 1995.

K. Mayahara and H. Ito, “Readmission of discharged schizophrenic patients with and without day care in Japan,” *Int.Med.J.*, vol. 9, no. 2, pp. 121–123, 2002.

L. Mellesdal, L. Mehlum, T. Wentzel-Larsen, R. Kroken, and H. A. Jørgensen, “Suicide risk and acute psychiatric readmissions: A prospective cohort study,” *Psychiatric Services*, vol. 61, no. 1, pp. 25–31, 2010.

D. J. Merchant and P. A. Henfling, “Scheduled brief admissions: patient ‘tuneups’.,” *Journal of Psychosocial Nursing & Mental Health Services*, vol. 32, no. 12, pp. 7–10, 1994.

E. L. Merrick, “Effects of a behavioral health carve-out on inpatient-related quality indicators for major depression treatment.,” *Med.Care*, vol. 37, no. 10, pp. 1023–1033, 1999.

J. R. Mertens, C. M. Weisner, and G. T. Ray, “Readmission among chemical dependency patients in private, outpatient treatment: patterns, correlates and role in long-term outcome.,” *J.Stud.Alcohol*, vol. 66, no. 6, pp. 842–847, 2005.

G. S. Mesch and G. Fishman, “First readmission of the mentally ill: An event history analysis,” *Soc.Sci.Res.*, vol. 23, no. 4, pp. 295–314, 1994.

T. Mgutshini, “Risk factors for psychiatric re-hospitalization: an exploration.,” *International Journal of Mental Health Nursing*, vol. 19, no. 4, pp. 257–267, 2010.

R. Mojtabai, R. A. Nicholson, and D. H. Neesmith, “Factors affecting relapse in patients discharged from a public hospital: results from survival analysis.,” *Psychiatr.Q.*, vol. 68, no. 2, pp. 117–129, 1997.

E. P. Monnelly, “Instability before discharge and previous psychiatric admissions as predictors of early readmission.,” *Psychiatric Services*, no. Journal Article, 1997.

C. M. Monson, D. D. Gunnin, M. H. Fogel, and L. L. Kyle, “Stopping (or slowing) the revolving door: factors related to NGRI acquittees’ maintenance of a conditional release.,” *Law & Human Behavior*, vol. 25, no. 3, pp. 257–267, 2001.

R. H. Moos, P. L. Brennan, and J. R. Mertens, “Diagnostic subgroups and predictors of one-year re-admission among late-middle-aged and older substance abuse patients.,” *J.Stud.Alcohol*, vol. 55, no. 2, pp. 173–183, 1994.

R. H. Moos, J. R. Mertens, and P. L. Brennan, “Program characteristics and readmission among older substance abuse patients: comparisons with middle-aged and younger patients.,” *J.Ment.Health Adm.*, vol. 22, no. 4, pp. 332–345, 1995.

R. H. Moos and B. S. Moos, “Stay in residential facilities and mental health care as predictors of readmission for patients with substance use disorders.,” *Psychiatric Services*, no. Journal Article, 1995.

P. W. Moran, L. A. Doerfler, J. Scherz, and J. D. Lish, “Rehospitalization of psychiatric patients in a managed care environment,” *Ment.Health Serv.Res.*, vol. 2, no. 4, pp. 191–198, 2000.

D. L. Mori and D. D. Blake, “Behavioral consultation with difficult to treat psychiatric patients,” *Percept.Mot.Skills*, vol. 74, no. 3, pp. 727–736, 1992.

G. Morken, J. H. Widen, and R. W. Grawe, “Non-adherence to antipsychotic medication, relapse and rehospitalisation in recent-onset schizophrenia.,” *BMC Psychiatry*, vol. 8, no. Journal Article, p. 32, 2008.

M. Morlino, A. Calento, V. Schiavone, G. Santone, A. Picardi, G. de Girolamo, and group PROGRES-Acute, “Use of psychiatric inpatient services by heavy users: findings from a national survey in Italy.,” *European Psychiatry: the Journal of the Association of European Psychiatrists*, vol. 26, no. 4, pp. 252–259, 2011.

P. B. Mortensen and W. W. Eaton, “Predictors for readmission risk in schizophrenia.,” *Psychol.Med.*, vol. 24, no. 1, pp. 223–232, 1994.

P. Mozny and P. Votypkova, “Expressed emotion, relapse rate and utilization of psychiatric inpatient care in schizophrenia. A study from Czechoslovakia.,” *Social Psychiatry & Psychiatric Epidemiology*, vol. 27, no. 4, pp. 174–179, 1992.

P. Muller, H. Nerenz, and E. Schaefer, “[The risk of rehospitalisation during therapy with atypical and typical neuroleptics--a contribution to differential indication],” *Psychiatr.Prax.*, vol. 29, no. 7, pp. 388–391, 2002.

W. Muller-Clemm, “Halting the ‘revolving door’ of serious mental illness: Evaluating an assertive case management program,” ProQuest Information & Learning, US, 1998.

T. Munk-Olsen, T. M. Laursen, C. B. Pedersen, O. Lidegaard, and P. B. Mortensen, “First-time first-trimester induced abortion and risk of readmission to a psychiatric hospital in women with a history of treated mental disorder.,” *Arch.Gen.Psychiatry*, vol. 69, no. 2, pp. 159–165, 2012.

A. Nager, R. Szulkin, S. E. Johansson, L. M. Johansson, and K. Sundquist, “High lifelong relapse rate of psychiatric disorders among women with postpartum psychosis.,” *Nordic Journal of Psychiatry*, vol. 67, no. 1, pp. 53–58, 2013.

K. Naka and S. Inoue, “Significant predictors for readmission of the severely mentally ill to a provincial hospital: is the efficacy of community care explicit or implicit?.,” *Japanese Journal of Psychiatry & Neurology*, vol. 48, no. 1, pp. 49–56, 1994.

A. Nazir, M. LaMantia, J. Chodosh, B. Khan, N. Campbell, S. Hui, and M. Boustani, “Interaction between cognitive impairment and discharge destination and its effect on rehospitalization.,” *J.Am.Geriatr.Soc.*, vol. 61, no. 11, pp. 1958–1963, 2013.

E. A. Nelson, M. E. Maruish, and J. L. Axler, “Effects of discharge planning and compliance with outpatient appointments on readmission rates.,” *Psychiatric Services*, vol. 51, no. 7, pp. 885–889, 2000.

T. P. Ng, M. Niti, W. C. Tan, Z. Cao, K. C. Ong, and P. Eng, “Depressive symptoms and chronic obstructive pulmonary disease: effect on mortality, hospital readmission, symptom burden, functional status, and quality of life.,” *Arch.Intern.Med.*, vol. 167, no. 1, pp. 60–67, 2007.

D. J. Niehaus, L. Koen, U. Galal, K. Dhansay, P. P. Oosthuizen, R. A. Emsley, and E. Jordaan, “Crisis discharges and readmission risk in acute psychiatric male inpatients,” *BMC Psychiatry*, vol. 8, no. 1, p. 44, 2008.

B. Nielsen, A. Moltke, J. K. Larsen, and P. Grinsted, “[Fewer readmissions of schizophrenic patients who have contact with their own GP],” *Ugeskr.Laeger*, vol. 170, no. 47, pp. 3862–3866, 2008.

S. Niksalehi, M. Fallahi, A. Rahgo, M. Rahgozar, H. Khankeh, and M. Bamdad, “Comparison the impact of home care services and Telephone Follow up on Rehospitalization and Mental condition of Schizophrenic patients,” *Res J Biol Sci*, vol. 6, no. 9, pp. 440–445, 2011.

A. Nordenskjold, L. von Knorring, and I. Engstrom, “Rehospitalization rate after continued electroconvulsive therapy--a retrospective chart review of patients with severe depression.,” *Nordic Journal of Psychiatry*, vol. 65, no. 1, pp. 26–31, 2011.

J. Novacek and R. Raskin, “Recognition of warning signs: a consideration for cost-effective treatment of severe mental illness.,” *Psychiatric Services*, vol. 49, no. 3, pp. 376–378, 1998.

D. W. O’Connor, B. Gardner, I. Presnell, D. Singh, M. Tsanglis, and E. White, “The effectiveness of continuation-maintenance ECT in reducing depressed older patients’ hospital re-admissions.,” *J.Affect.Disord.*, vol. 120, no. 1–3, pp. 62–66, 2010.

H. Odes, N. Katz, E. Noter, Y. Shamir, A. Weizman, and A. Valevski, “Level of function at discharge as a predictor of readmission among inpatients with schizophrenia.,” *American Journal of Occupational Therapy*, vol. 65, no. 3, pp. 314–319, 2011.

B. O’Donoghue, J. Lyne, M. Hill, L. O’Rourke, S. Daly, C. Larkin, L. Feeney, and E. O’Callaghan, “Perceptions of involuntary admission and risk of subsequent readmission at one-year follow-up: the influence of insight and recovery style.,” *Journal of Mental Health*, vol. 20, no. 3, pp. 249–259, 2011.

R. O. Ogedengbe, “Some contributions of the traditional psychiatrists toward mental health in Nigeria,” *IFE Psychologia: An International Journal*, vol. 1, no. 2, pp. 17–31, 1993.

T. Oiesvold, O. Saarento, S. Sytema, H. Vinding, G. Gostas, O. Lonnerberg, S. Muus, M. Sandlund, and L. Hansson, “Predictors for readmission risk of new patients: the Nordic Comparative Study on Sectorized Psychiatry.,” *Acta Psychiatr.Scand.*, vol. 101, no. 5, pp. 367–373, 2000.

D. A. O’Leary and A. S. Lee, “Seven year prognosis in depression. Mortality and readmission risk in the Nottingham ECT cohort.,” *British Journal of Psychiatry*, vol. 169, no. 4, pp. 423–429, 1996.

A. V. Olesen and P. B. Mortensen, “Readmission risk in schizophrenia: selection explains previous findings of a progressive course of disorder.,” *Psychol.Med.*, vol. 32, no. 7, pp. 1301–1307, 2002.

M. Olfson, D. Mechanic, C. A. Boyer, S. Hansell, J. Walkup, and P. J. Weiden, “Assessing clinical predictions of early rehospitalization in schizophrenia,” *J.Nerv.Ment.Dis.*, vol. 187, no. 12, pp. 721–729, 1999.

T. Ono, A. Tamai, D. Takeuchi, and Y. Tamai, “Factors related to readmission to a ward for dementia patients: Sex differences,” *Psychiatry Clin.Neurosci.*, vol. 65, no. 5, pp. 490–498, 2011.

C. Owen, V. Rutherford, M. Jones, C. Tennant, and A. Smallman, “Psychiatric rehospitalization following hospital discharge,” *Community Ment.Health J.*, vol. 33, no. 1, pp. 13–24, 1997.

I. Oyffe, R. Kurs, M. Gelkopf, Y. Melamed, and A. Bleich, “Revolving-door patients in a public psychiatric hospital in Israel: Cross sectional study,” *Croat.Med.J.*, vol. 50, no. 6, pp. 575–582, 2009.

A. Papageorgiou, M. King, A. Janmohamed, O. Davidson, and J. Dawson, “Advance directives for patients compulsorily admitted to hospital with serious mental illness. Randomised controlled trial.,” *British Journal of Psychiatry*, vol. 181, no. Journal Article, pp. 513–519, 2002.

G. Parker and D. Hadzi-Pavlovic, “The capacity of a measure of disability (the LSP) to predict hospital readmission in those with schizophrenia.,” *Psychol.Med.*, vol. 25, no. 1, pp. 157–163, 1995.

G. B. Parker, C. A. Owen, H. L. Brotchie, and M. P. Hyett, “The impact of differing anxiety disorders on outcome following an acute coronary syndrome: time to start worrying?.,” *Depression & Anxiety*, vol. 27, no. 3, pp. 302–309, 2010.

C. D. Parry and E. Turkheimer, “Length of hospitalization and outcome of commitment and recommitment hearings.,” *Hosp.Community Psychiatry*, vol. 43, no. 1, pp. 65–68, 1992.

N. C. Patel, M. L. Crismon, and M. Pondrom, “Rehospitalization rates of patients with bipolar disorder discharged on a mood stabilizer versus a mood stabilizer plus an atypical or typical antipsychotic.,” *J.Behav.Health Serv.Res.*, vol. 32, no. 4, pp. 438–445, 2005.

N. C. Patel, P. G. Dorson, N. Edwards, S. Mendelson, and M. L. Crismon, “One-year rehospitalization rates of patients discharged on atypical versus conventional antipsychotics.,” *Psychiatric Services*, vol. 53, no. 7, pp. 891–893, 2002.

D. A. Patterson and R. N. Cloud, “The application of artificial neural networks for outcome prediction in a cohort of severely mentally ill outpatients,” *Journal of Technology in Human Services*, vol. 16, no. 2–3, pp. 47–61, 1999.

D. A. Patterson and M.-S. Lee, “Intensive case management and rehospitalization: A survival analysis,” *Research on Social Work Practice*, vol. 8, no. 2, pp. 152–171, 1998.

J. Pedersen and T. Aarkrog, “A 10-year follow-up study of an adolescent psychiatric clientele and early predictors of readmission,” *Nordic Journal of Psychiatry*, vol. 55, no. 1, pp. 11–16, 2001.

E. F. Perese, “Unmet needs of persons with chronic mental illnesses: Relationship to their adaptation to community living,” *Issues Ment.Health Nurs.*, vol. 18, no. 1, pp. 19–34, 1997.

K. A. Peterson, R. W. Swindle, C. S. Phibbs, B. Recine, and R. H. Moos, “Determinants of readmission following inpatient substance abuse treatment: a national study of VA programs.,” *Med.Care*, vol. 32, no. 6, pp. 535–550, 1994.

P. N. Pfeiffer, D. Ganoczy, K. Zivin, J. F. McCarthy, M. Valenstein, and F. C. Blow, “Outpatient follow-up after psychiatric hospitalization for depression and later readmission and treatment adequacy,” *Psychiatric Services*, vol. 63, no. 12, pp. 1239–1242, 2012.

C. S. Phibbs, R. W. Swindle, and B. Recine, “Does case mix matter for substance abuse treatment? A comparison of observed and case mix-adjusted readmission rates for inpatient substance abuse treatment in the Department of Veterans Affairs.,” *Health Serv.Res.*, vol. 31, no. 6, pp. 755–771, 1997.

A. L. Pillay, W. F. du Plessis, N. B. M. Vawda, and L. R. Pollock, “Demographic and readmission data in a therapeutic community for Black psychiatric patients in South Africa,” *Aust.N.Z.J.Psychiatry*, vol. 28, no. 4, pp. 684–688, 1994.

S. Pollack, M. G. Woerner, A. Howard, R. B. Fireworker, and J. M. Kane, “Clozapine reduces rehospitalization among schizophrenia patients.,” *Psychopharmacol.Bull.*, vol. 34, no. 1, pp. 89–92, 1998.

K. Pono, “Association of length of stay and readmission rate among schizophrenic patients in NakhonPhanom Psychiatric Hospital, Thailand,” 2009.

S. Ponzer, S. E. Johansson, and B. Bergman, “A four-year follow-up study of male alcoholics: factors affecting the risk of readmission.,” *Alcohol*, vol. 27, no. 2, pp. 83–88, 2002.

T. T. Postolache, P. B. Mortensen, L. H. Tonelli, X. Jiao, C. Frangakis, J. J. Soriano, and P. Qin, “Seasonal spring peaks of suicide in victims with and without prior history of hospitalization for mood disorders.,” *J.Affect.Disord.*, vol. 121, no. 1–2, pp. 88–93, 2010.

S. Pridmore, H. Hornsby, D. Hay, and I. Jones, “Survival analysis and readmission in mood disorder,” *The British Journal of Psychiatry*, vol. 165, no. 6, pp. 824–827, 1994.

J. D. Prince, A. Akincigil, E. Kalay, J. T. Walkup, D. R. Hoover, J. Lucas, J. Bowblis, and S. Crystal, “Psychiatric rehospitalization among elderly persons in the United States.,” *Psychiatric Services*, vol. 59, no. 9, pp. 1038–1045, 2008.

J. D. Prince, “Practices Preventing Rehospitalization of Individuals With Schizophrenia,” *J.Nerv.Ment.Dis.*, vol. 194, no. 6, pp. 397–403, 2006.

J. Rabinowitz, P. Lichtenberg, Z. Kaplan, M. Mark, D. Nahon, and M. Davidson, “Rehospitalization rates of chronically ill schizophrenic patients discharged on a regimen of risperidone, olanzapine, or conventional antipsychotics.,” *Am.J.Psychiatry*, vol. 158, no. 2, pp. 266–269, 2001.

E. Rameshwar, “A mixed method approach investigating the‘ revolving door’ patient with severe mental health problems,” 2004.

J. I. Ramirez, “Prediction of psychiatric rehospitalization among a predominantly Hispanic population receiving services at a community mental health center,” 2000.

D. K. R. Ramos and J. Guimarães, “Novos serviços de saúde mental eo fenômeno da porta giratória no Rio Grande do Norte; New mental health services and the revolving door phenomenon in Rio Grande do Norte,” *REME rev.min.enferm*, vol. 17, no. 2, pp. 198–203, 2013.

W. Reynolds, W. Lauder, S. Sharkey, S. MacIver, T. Veitch, and D. Cameron, “The effects of a transitional discharge model for psychiatric patients,” *J.Psychiatr.Ment.Health Nurs.*, vol. 11, no. 1, pp. 82–88, 2004.

D. E. Richardson, “The Relationships Among Global Assessment Functioning, Length of Stay, Readmission, and Mental Health Intensive Case Management in Veterans,” 2002.

P. D. Riley, “The impact of co-treating disciplines on length of stay and readmission in a private psychiatric hospital,” ProQuest Information & Learning, US, 1997.

S. Riordan, S. Haque, and M. Humphreys, “Possible predictors of outcome for conditionally discharged patients--a preliminary study.,” *Medicine, Science & the Law*, vol. 46, no. 1, pp. 31–36, 2006.

H. Rittmannsberger, T. Pachinger, P. Keppelmüller, and J. Wancata, “Medication adherence among psychotic patients before admission to inpatient treatment,” *Psychiatric Services*, vol. 55, no. 2, pp. 174–179, 2004.

F. P. Rivara, T. D. Koepsell, G. J. Jurkovich, J. G. Gurney, and R. Soderberg, “The effects of alcohol abuse on readmission for trauma.,” *JAMA*, vol. 270, no. 16, pp. 1962–1964, 1993.

A. Romelsjo, T. Palmstierna, H. Hansagi, and A. Leifman, “Length of outpatient addiction treatment and risk of rehospitalization.,” *J.Subst.Abuse Treat.*, vol. 28, no. 3, pp. 291–296, 2005.

P. Rosca, A. Bauer, A. Grinshpoon, R. Khawaled, R. Mester, and A. M. Ponizovsky, “Rehospitalizations Among Psychiatric Patients Whose First Admission was Involuntary: A 10-Year Follow-Up,” *Isr.J.Psychiatry Relat.Sci.*, vol. 43, no. 1, pp. 57–64, 2006.

S. Rosenfield, “Homelessness and rehospitalization: The importance of housing for the chronic mentally ill,” *J.Community Psychol.*, vol. 19, no. 1, pp. 60–69, 1991.

R. Rosenheck and M. Neale, “Intersite variation in the impact of intensive psychiatric community care on hospital use.,” *Am.J.Orthopsychiatry*, vol. 68, no. 2, pp. 191–200, 1998.

W. Rossler, W. Loffler, B. Fatkenheuer, and A. Riecher-Rossler, “Does case management reduce the rehospitalization rate?.,” *Acta Psychiatr.Scand.*, vol. 86, no. 6, pp. 445–449, 1992.

W. Rossler, W. Loffler, B. Fatkenheuer, and A. Riecher-Rossler, “Case management for schizophrenic patients at risk for rehospitalization: a case control study.,” *European Archives of Psychiatry & Clinical Neuroscience*, vol. 246, no. 1, pp. 29–36, 1995.

A. B. Rothbard, S. Chhatre, C. Zubritsky, K. Fortuna, S. Dettwyler, R. J. Henry, and M. Smith, “Effectiveness of a high end users program for persons with psychiatric disorders.,” *Community Ment.Health J.*, vol. 48, no. 5, pp. 598–603, 2012.

P. Ruesch, P. C. Meyer, and D. Hell, “[Who is rehospitalized in a psychiatric hospital? Psychiatric hospitalization rates and social indicators in the Zurich canton (Switzerland)],” *Gesundheitswesen*, vol. 62, no. 3, pp. 166–171, 2000.

J. Russo, P. Roy-Byrne, C. Jaffe, R. Ries, C. Dagadakis, and D. Avery, “Psychiatric status, quality of life, and level of care as predictors of outcomes of acute inpatient treatment.,” *Psychiatric Services*, vol. 48, no. 11, pp. 1427–1434, 1997.

O. Saarento, P. Nieminen, H. Hakko, M. Isohanni, and E. Väisänen, “Utilization of psychiatric in-patient care among new patients in a comprehensive community-care system: A 3-year follow-up study,” *Acta Psychiatr.Scand.*, vol. 95, no. 2, pp. 132–139, 1997.

M. M. Salles and S. Barros, “[Readmission to a psychiatric hospital: the comprehension of the health/illness process through the experience of daily life],” *Revista Da Escola de Enfermagem Da Usp*, vol. 41, no. 1, pp. 73–81, 2007.

R. Sanchez, L. E. Jaramillo, and M. I. Herazo, “[Factors associated with early psychiatric rehospitalization],” *Biomedica*, vol. 33, no. 2, pp. 276–282, 2013.

V. R. Sanguineti, S. E. Samuel, S. L. Schwartz, and M. R. Robeson, “Retrospective study of 2,200 involuntary psychiatric admissions and readmissions.,” *Am.J.Psychiatry*, vol. 153, no. 3, pp. 392–396, 1996.

S. M. Saravay, S. Pollack, M. D. Steinberg, B. Weinschel, and M. Habert, “Four-year follow-up of the influence of psychological comorbidity on medical rehospitalization,” *Am.J.Psychiatry*, vol. 153, no. 3, pp. 397–403, 1996.

N. Satake, “[Antipsychotic medication change and reduction of rehospitalization in clients of ACT-J],” *Seishin Shinkeigaku Zasshi - Psychiatria et Neurologia Japonica*, vol. 113, no. 6, pp. 612–618, 2011.

R. L. Schalock, F. Touchstone, G. Nelson, L. Weber, M. Sheehan, and C. Stull, “A multivariate analysis of mental hospital recidivism.,” *J.Ment.Health Adm.*, vol. 22, no. 4, pp. 358–367, 1995.

C. Schmidt-Kraepelin, B. Janssen, and W. Gaebel, “Prevention of rehospitalization in schizophrenia: results of an integrated care project in Germany.,” *European Archives of Psychiatry & Clinical Neuroscience*, vol. 259, no. Suppl 2, pp. S205–12, 2009.

S. C. Schoenbaum, D. Cookson, and S. Stelovich, “Postdischarge follow-up of psychiatric inpatients and readmission in an HMO setting,” *Psychiatric Services*, vol. 46, no. 9, pp. 943–945, 1995.

N. R. Schooler, S. J. Keith, J. B. Severe, S. M. Matthews, A. S. Bellack, I. D. Glick, W. A. Hargreaves, J. M. Kane, P. T. Ninan, A. Frances, M. Jacobs, J. A. Lieberman, R. Mance, G. M. Simpson, and M. G. Woerner, “Relapse and rehospitalization during maintenance treatment of schizophrenia. The effects of dose reduction and family treatment.,” *Arch.Gen.Psychiatry*, vol. 54, no. 5, pp. 453–463, 1997.

J. Scott and M. Pope, “Self-reported adherence to treatment with mood stabilizers, plasma levels, and psychiatric hospitalization.,” *Am.J.Psychiatry*, vol. 159, no. 11, pp. 1927–1929, 2002.

S. P. Segal, P. D. Akutsu, and M. A. Watson, “Factors associated with involuntary return to a psychiatric emergency service within 12 months.,” *Psychiatric Services*, vol. 49, no. 9, pp. 1212–1217, 1998.

S. P. Segal, P. D. Akutsu, and M. A. Watson, “Involuntary return to a psychiatric emergency service within twelve months.,” *Soc.Work Health Care*, vol. 35, no. 1–2, pp. 591–603, 2002.

K. L. Serowik and P. Yanos, “The relationship between services and outcomes for a prison reentry population of those with severe mental illness,” *Mental Health and Substance Use*, vol. 6, no. 1, pp. 4–14, 2013.

A. Shaner, T. A. Eckman, L. J. Roberts, J. N. Wilkins, D. E. Tucker, J. W. Tsuang, and J. Mintz, “Disability income, cocaine use, and repeated hospitalization among schizophrenic cocaine abusers--a government-sponsored revolving door?.,” *N.Engl.J.Med.*, vol. 333, no. 12, pp. 777–783, 1995.

G. Shankar, “Impact of psychiatric hospital readmission rates through pharmacist intervention-a retrospectve analysis.,” presented at the PHARMACOTHERAPY, 2013, vol. 33, pp. E299–E299.

W. Shao, H. Chen, Y. Chang, W. Lin, and E. Lin, “[The Relationship Between Medication Adherence and Rehospitalization: A Prospective Study of Schizophrenia Patients Discharged From Psychiatric Acute Wards.],” *Hu Li Za Zhi*, vol. 60, no. 5, pp. 31–40, 2013.

V. Sharifi, M. Tehranidoost, M. Yunesian, H. Amini, M. Mohammadi, and M. Jalali Roudsari, “Effectiveness of a low-intensity home-based aftercare for patients with severe mental disorders: a 12-month randomized controlled study.,” *Community Ment.Health J.*, vol. 48, no. 6, pp. 766–770, 2012.

C. Shea, “A monitored medication compliance program and its effect on relapse and readmission for the psychiatric client,” 1992.

M. C. Silva and M. C. Stefanelli, “[A preliminary study of the factors which lead to patient readmission in psychiatric hospitals],” *Rev.Paul.Enferm.*, vol. 10, no. 1, pp. 21–28, 1991.

N. C. Silva, D. G. Bassani, and L. S. Palazzo, “A case-control study of factors associated with multiple psychiatric readmissions,” *Psychiatric Services*, vol. 60, no. 6, pp. 786–791, 2009.

M. L. Silverstein, L. Fogg, and M. Harrow, “Prognostic significance of cerebral status: Dimensions of clinical outcome,” *J.Nerv.Ment.Dis.*, vol. 179, no. 9, pp. 534–539, 1991.

H. Singh, D. A. Bhalchandra, S. Sarmukaddam, and S. K. Chaturvedi, “Readmission of psychiatric patients in India: sociodemographic factors,” *International Journal of Culture and Mental Health*, no. ahead-of-print, pp. 1–12, 2013.

W. B. Slate, “The Relationship Between Family Teaching and Rehospitalization in an Acute Psychiatric Setting,” 2003.

W. H. Sledge, M. Lawless, D. Sells, M. Wieland, M. J. O’Connell, and L. Davidson, “Effectiveness of peer support in reducing readmissions of persons with multiple psychiatric hospitalizations.,” *Psychiatric Services*, vol. 62, no. 5, pp. 541–544, 2011.

H. M. Smith, “Factors leading to frequent readmission to Valkenberg Hospital for patients suffering from severe mental illnesses.,” 2005.

M. Smith and S. K. Schultz, “Managing perplexing patients: the case of Helen.,” *Issues Ment.Health Nurs.*, vol. 26, no. 1, pp. 47–63, 2005.

R. Smith, P. De Witt, D. Franzsen, M. Pilley, N. Wolfe, and C. Davies, “Occupational performance factors perceived to influence the readmission of mental health care users diagnosed with schizophrenia,” *South African Journal of Occupational Therapy*, vol. 44, no. 1, pp. 51–56, 2014.

S. L. Smoot, R. M. Vandiver, and R. A. Fields, “Homeless persons readmitted to an urban state hospital.,” *Hosp.Community Psychiatry*, vol. 43, no. 10, pp. 1028–1030, 1992.

M. B. Snowden, A. Walaszek, J. E. Russo, K. A. Comtois, D. S. Srebnik, R. K. Ries, and P. Roy-Byrne, “Geriatric Patients Improve as Much as Younger Patients from Hospitalization on General Psychiatric Units,” *J.Am.Geriatr.Soc.*, vol. 52, no. 10, pp. 1676–1680, 2004.

S. D. Soni, K. Gaskell, and P. Reed, “Factors affecting rehospitalisation rates of chronic schizophrenic patients living in the community,” *Schizophr.Res.*, vol. 12, no. 2, pp. 169–177, 1994.

M. Soyka, B. Helten, M. Cleves, and P. Schmidt, “High rehospitalization rate in alcohol-induced psychotic disorder,” *Eur.Arch.Psychiatry Clin.Neurosci.*, vol. 263, no. 4, pp. 309–313, 2013.

H. Spiessl, B. Hubner-Liebermann, H. Binder, and C. Cording, “[Heavy users in a psychiatric hospital--a cohort study on 1811 patients over five years],” *Psychiatr.Prax.*, vol. 29, no. 7, pp. 350–354, 2002.

G. J. Stahler, J. Mennis, R. Cotlar, and D. A. Baron, “The influence of neighborhood environment on treatment continuity and rehospitalization in dually diagnosed patients discharged for acute inpatient care,” *Am.J.Psychiatry*, vol. 166, no. 11, pp. 1258–1268, 2009.

J. H. Starrfield, M. Avnon, W. Starrfield, J. Rabinowitz, and S. Heifetz, “Effects of psychosocial rehabilitation for hospitalized mentally ill homeless persons,” *Psychiatric Services*, vol. 46, no. 9, pp. 948–950, 1995.

M. Startup, M. C. Jackson, and S. Startup, “Insight, social functioning and readmission to hospital in patients with schizophrenia-spectrum disorders: prospective associations.,” *Psychiatry Res.*, vol. 178, no. 1, pp. 17–22, 2010.

T. Steinert, C. Wiebe, and R.-P. Gebhardt, “Geht fremd- und selbstgerichtetes aggressives Verhalten bei schizophrenen Patienten mit ungünstigeren Krankheitsverläufen einher? = Is aggressive behaviour against others and self associated with an unfavorable course of illness in schizophrenics?,” *Krankenhauspsychiatrie*, vol. 10, no. 2, pp. 45–49, 1999.

T. Steinert, C. Wiebe, and R. P. Gebhardt, “Aggressive behavior against self and others among first-admission patients with schizophrenia,” *Psychiatric Services*, vol. 50, no. 1, pp. 85–90, 1999.

H. C. Steinhausen, M. Grigoroiu-Serbanescu, S. Boyadjieva, K. J. Neumarker, and C. Winkler Metzke, “Course and predictors of rehospitalization in adolescent anorexia nervosa in a multisite study.,” *Int.J.Eat.Disord.*, vol. 41, no. 1, pp. 29–36, 2008.

V. Stieffenhofer, H. Saglam, I. Schmidtmann, H. Silver, C. Hiemke, and A. Konrad, “Clozapine plasma level monitoring for prediction of rehospitalization schizophrenic outpatients.,” *Pharmacopsychiatry*, vol. 44, no. 2, pp. 55–59, 2011.

A. Stoudemire, C. D. Hill, S. T. Dalton, and M. G. Marquardt, “Rehospitalization rates in older depressed adults after antidepressant and electroconvulsive therapy treatment.,” *J.Am.Geriatr.Soc.*, vol. 42, no. 12, pp. 1282–1285, 1994.

O. Strasser, M. Schmauss, and T. Messer, “[Rehospitalization rates of newly diagnosed schizophrenic patients on atypical neuroleptic medication],” *Psychiatr.Prax.*, vol. 31, no. Suppl 1, pp. S38–40, 2004.

S. S. Sulaiman and K. K. Malaysia, “The Impact of psychiatry services on readmission and maitaining contact of psychiatric care in Alor Setar,” no. Journal Article, 2007.

G. Sullivan, A. S. Young, and H. Morgenstern, “Behaviors as risk factors for rehospitalization: Implications for predicting and preventing admissions among the seriously mentally ill,” *Soc.Psychiatry Psychiatr.Epidemiol.*, vol. 32, no. 4, pp. 185–190, 1997.

G. Sullivan, K. B. Wells, H. Morgenstern, and B. Leake, “Identifying modifiable risk factors for rehospitalization: A case-control study of seriously mentally ill persons in Mississippi,” *Am.J.Psychiatry*, vol. 152, no. 12, pp. 1749–1756, 1995.

Y. Suzuki, S. Yasumura, A. Fukao, and K. Otani, “Associated factors of rehospitalization among schizophrenic patients.,” *Psychiatry & Clinical Neurosciences*, vol. 57, no. 6, pp. 555–561, 2003.

M. S. Swartz, J. W. Swanson, H. R. Wagner, B. J. Burns, V. A. Hiday, and R. Borum, “Can involuntary outpatient commitment reduce hospital recidivism?: Findings from a randomized trial with severely mentally ill individuals,” *Am.J.Psychiatry*, vol. 156, no. 12, pp. 1968–1975, 1999.

C. Swett, “Symptom severity and number of previous psychiatric admissions as predictors of readmission,” *Psychiatric Services*, vol. 46, no. 5, pp. 482–485, 1995.

R. W. Swindle, C. S. Phibbs, M. J. Paradise, B. P. Recine, and R. H. Moos, “Inpatient treatment for substance abuse patients with psychiatric disorders: A national study of determinants of readmission,” *J.Subst.Abuse*, vol. 7, no. 1, pp. 79–97, 1995.

S. Sytema, P. Burgess, and M. Tansella, “Does community care decrease length of stay and risk of rehospitalization in new patients with schizophrenia disorders? A comparative case register study in Groningen, The Netherlands; Victoria, Australia; and South-Verona, Italy.,” *Schizophr.Bull.*, vol. 28, no. 2, pp. 273–281, 2002.

S. Sytema and P. Burgess, “Continuity of care and readmission in two service systems: a comparative Victorian and Groningen case-register study.,” *Acta Psychiatr.Scand.*, vol. 100, no. 3, pp. 212–219, 1999.

R. Tavcar, M. Z. Dernovsek, and V. Zvan, “Choosing antipsychotic maintenance therapy–A naturalistic study,” *Pharmacopsychiatry*, vol. 33, no. 2, pp. 66–71, 2000.

I. M. Terp, G. Engholm, H. Moller, and P. B. Mortensen, “A follow-up study of postpartum psychoses: prognosis and risk factors for readmission.,” *Acta Psychiatr.Scand.*, vol. 100, no. 1, pp. 40–46, 1999.

N. M. Thakur, R. A. Hoff, B. Druss, and J. Catalanotto, “Using recidivism rates as a quality indicator for substance abuse treatment programs.,” *Psychiatric Services*, vol. 49, no. 10, pp. 1347–1350, 1998.

M. R. Thomas, S. A. Rosenberg, A. A. Giese, and G. E. Fryer, “Shortening length of stay without increasing recidivism on a university-affiliated inpatient unit,” *Psychiatric Services*, vol. 47, no. 9, pp. 996–998, 1996.

E. E. Thompson, H. W. Neighbors, C. Munday, and S. Trierweiler, “Length of Stay, Referral to Aftercare, and Rehospitalization Among Psychiatric Inpatients,” *Psychiatric Services*, vol. 54, no. 9, pp. 1271–1276, 2003.

P. H. Thomsen, “A 22- to 25-year follow-up study of former child psychiatric patients: A register-based investigation of the course of psychiatric disorder and mortality in 546 Danish child psychiatric patients,” *Acta Psychiatr.Scand.*, vol. 94, no. 6, pp. 397–403, 1996.

P. H. Thomsen, “The prognosis in early adulthood of child psychiatric patients: a case register study in Denmark.,” *Acta Psychiatr.Scand.*, vol. 81, no. 1, pp. 89–93, 1990.

G. Thornicroft, C. Gooch, and D. Dayson, “The TAPS project. 17: Readmission to hospital for long term psychiatric patients after discharge to the community.,” *BMJ*, vol. 305, no. 6860, pp. 996–998, 1992.

J. Tiihonen, J. Haukka, M. Taylor, P. M. Haddad, M. X. Patel, and P. Korhonen, “A nationwide cohort study of oral and depot antipsychotics after first hospitalization for schizophrenia,” *Am.J.Psychiatry*, vol. 168, no. 6, pp. 603–609, 2011.

K. Tómasson and P. Vaglum, “The role of psychiatric comorbidity in the prediction of readmission for detoxification,” *Compr.Psychiatry*, vol. 39, no. 3, pp. 129–136, 1998.

A. Tomita and D. B. Herman, “The impact of critical time intervention in reducing psychiatric rehospitalization after hospital discharge,” *Psychiatric Services*, vol. 63, no. 9, pp. 935–937, 2012.

A. Tomita, E. P. Lukens, and D. B. Herman, “Mediation analysis of critical time intervention for persons living with serious mental illnesses: Assessing the role of family relations in reducing psychiatric rehospitalization,” *Psychiatr.Rehabil.J.*, vol. 37, no. 1, pp. 4–10, 2014.

G. Torisson, L. Minthon, L. Stavenow, and E. Londos, “Multidisciplinary intervention reducing readmissions in medical inpatients: a prospective, non-randomized study.,” *Clinical Interventions In Aging*, vol. 8, no. Journal Article, pp. 1295–1304, 2013.

J. L. Toth, “The role of selected personality characteristics in the prediction of psychiatric rehospitalization: deinstitutionalization re-visited,” 1992.

J. Trujols, J. Guardia, M. Pero, M. Freixa, N. Sinol, A. Tejero, and J. Perez de Los Cobos, “Multi-episode survival analysis: an application modelling readmission rates of heroin dependents at an inpatient detoxification unit.,” *Addict.Behav.*, vol. 32, no. 10, pp. 2391–2397, 2007.

A. Valevski, Y. Gilat, M. Olfson, N. Benaroya-Milshtein, and A. Weizman, “Antipsychotic monotherapy and adjuvant psychotropic therapies in schizophrenia patients: effect on time to readmission.,” *Int.Clin.Psychopharmacol.*, vol. 27, no. 3, pp. 159–164, 2012.

A. Valevski, G. Zalsman, S. Tsafrir, R. Lipschitz-Elhawi, A. Weizman, and T. Shohat, “Rate of readmission and mortality risks of schizophrenia patients who were discharged against medical advice.,” *European Psychiatry: the Journal of the Association of European Psychiatrists*, vol. 27, no. 7, pp. 496–499, 2012.

A. Valevski, M. Olfson, A. Weizman, and R. Shiloh, “Risk of readmission in compulsorily and voluntarily admitted patients,” *Soc.Psychiatry Psychiatr.Epidemiol.*, vol. 42, no. 11, pp. 916–922, 2007.

I. L. Vallejo, H. H. Herrero, J. J. C. Sanz, la G. de, and E. N. Azarola, “Variabilidad en los diagnósticos de una cohorte de reingresadores en las dos últimas décadas. = Diagnostic variability in a cohort of patients with multiple admissions in the last two decades,” *Actas Españolas de Psiquiatría*, vol. 31, no. 1, pp. 18–23, 2003.

S. Vasudeva, M. S. Narendra Kumar, and K. C. Sekhar, “Duration of first admission and its relation to the readmission rate in a psychiatry hospital.,” *Indian Journal of Psychiatry*, vol. 51, no. 4, pp. 280–284, 2009.

K. Vaughan, N. McConaghy, C. Wolf, C. Myhr, and T. Black, “Community treatment orders: Relationship to clinical care, medication compliance, behavioural disturbance and readmission,” *Aust.N.Z.J.Psychiatry*, vol. 34, no. 5, pp. 801–808, 2000.

H. Verdoux, F. Liraud, F. Assens, F. Abalan, and J. van Os, “Social and clinical consequences of cognitive deficits in early psychosis: A two-year follow-up study of first-admitted patients,” *Schizophr.Res.*, vol. 56, no. 1–2, pp. 148–159, 2002.

H. Verdoux, J. Van Os, P. Sham, P. Jones, K. Gilvarry, and R. Murray, “Does familiarity predispose to both emergence and persistence of psychosis? A follow-up study,” *The British Journal of Psychiatry*, vol. 168, no. 5, pp. 620–626, 1996.

P. Vetter and O. Koller, “Psychiatric and somatic hospitalisations of different diagnostic groups: a long-term follow-up study.,” *Psychopathology*, vol. 24, no. 6, pp. 381–387, 1991.

P. H. Vetter and O. Köller, “First hospitalized versus rehospitalized patients with an affective disorder or schizophrenia: Differences in long-term course and outcome,” *Psychopathology*, vol. 31, no. 5, pp. 260–264, 1998.

S. N. Vigod, V. H. Taylor, K. Fung, and P. A. Kurdyak, “Within-hospital readmission: An indicator of readmission after discharge from psychiatric hospitalization,” *The Canadian Journal of Psychiatry / La Revue canadienne de psychiatrie*, vol. 58, no. 8, pp. 476–481, 2013.

H. Villars, C. Dupuy, P. Soler, V. Gardette, M. E. Soto, S. Gillette, F. Nourhashemi, and B. Vellas, “A follow-up intervention in severely demented patients after discharge from a special Alzheimer acute care unit: impact on early emergency room re-hospitalization rate.,” *Int.J.Geriatr.Psychiatry*, vol. 28, no. 11, pp. 1131–1140, 2013.

S. Vogel and P. Huguelet, “Factors associated with multiple admissions to a public psychiatric hospital,” *Acta Psychiatr.Scand.*, vol. 95, no. 3, pp. 244–253, 1997.

R. Walker, D. Minor-Schork, R. Bloch, and J. Esinhart, “High risk factors for rehospitalization within six months,” *Psychiatr.Q.*, vol. 67, no. 3, pp. 235–243, 1996.

R. D. Walker, M. O. Howard, B. Anderson, P. S. Walker, M. D. Lambert, R. Suchinsky, and M. Johnson, “Diagnosis and hospital readmission rates of female veterans with substance-related disorders,” *Psychiatric Services*, vol. 46, no. 9, pp. 932–937, 1995.

S. A. Walker and J. M. Eagles, “Discharging psychiatric patients from hospital,” *Psychiatric Bulletin*, vol. 26, no. 7, pp. 241–242, 2002.

S. M. Walker, “Factors Affecting Violent Readmission to Hospital by Chronic Psychiatric Patients,” 1995.

A. Y. Walley, M. Paasche-Orlow, E. C. Lee, S. Forsythe, V. K. Chetty, S. Mitchell, and B. W. Jack, “Acute care hospital utilization among medical inpatients discharged with a substance use disorder diagnosis.,” *Journal of Addiction Medicine*, vol. 6, no. 1, pp. 50–56, 2012.

T. T. Wan and Y. A. Ozcan, “Determinants of psychiatric rehospitalization: a social area analysis,” *Community Ment.Health J.*, vol. 27, no. 1, pp. 3–16, 1991.

I. Warnke, C. Nordt, V. Ajdacic-Gross, A. Haug, H. J. Salize, and W. Rossler, “[Clinical and social risk factors for the readmission of patients with schizophrenia to psychiatric inpatient care: a long-term analysis],” *Neuropsychiatrie*, vol. 24, no. 4, pp. 243–251, 2010.

R. Warren, “Follow-up that reduces hospital readmissions of people with chronic mental illness.,” *Australian Journal of Advanced Nursing*, vol. 12, no. 2, pp. 26–31, 1994.

P. Weiden and W. Glazer, “Assessment and treatment selection for ‘revolving door’ inpatients with schizophrenia,” *Psychiatr.Q.*, vol. 68, no. 4, pp. 377–392, 1997.

G. Weithmann and M. Hoffmann, “[Frequency and costs of additional hospitalisations in the year after inpatient or day hospital detoxification treatment],” *Psychiatr.Prax.*, vol. 34, no. 1, pp. 15–19, 2007.

A. P. Werneck, J. C. Hallak, E. Nakano, and H. Elkis, “Time to rehospitalization in patients with schizophrenia discharged on first generation antipsychotics, non-clozapine second generation antipsychotics, or clozapine.,” *Psychiatry Res.*, vol. 188, no. 3, pp. 315–319, 2011.

A. Wheeler, S. Moyle, C. Jansen, E. Robinson, and J. Vanderpyl, “Five-year follow-up of an acute psychiatric admission cohort in Auckland, New Zealand.,” *N.Z.Med.J.*, vol. 124, no. 1336, pp. 30–38, 2011.

T. M. Wickizer and D. Lessler, “Do treatment restrictions imposed by utilization management increase the likelihood of readmission for psychiatric patients?,” *Med.Care*, vol. 36, no. 6, pp. 844–850, 1998.

I. M. Wieselgren and L. H. Lindstrom, “A prospective 1-5 year outcome study in first-admitted and readmitted schizophrenic patients; relationship to heredity, premorbid adjustment, duration of disease and education level at index admission and neuroleptic treatment.,” *Acta Psychiatr.Scand.*, vol. 93, no. 1, pp. 9–19, 1996.

K. Wilson, P. Mottram, and M. Hussain, “Survival in the community of the very old depressed, discharged from medical inpatient care.,” *Int.J.Geriatr.Psychiatry*, vol. 22, no. 10, pp. 974–979, 2007.

A. Winston, H. Pardes, D. S. Papernik, and L. Breslin, “Aftercare of Psychiatric Patients and Its Relation to Rehospitalization,” *Psychiatric Services*, no. Journal Article, 1997.

B. K. P. Woo, S. Golshan, E. C. Allen, J. W. Daly, D. V. Jeste, and D. D. Sewell, “Factors Associated With Frequent Admissions to an Acute Geriatric Psychiatric Inpatient Unit,” *J.Geriatr.Psychiatry Neurol.*, vol. 19, no. 4, pp. 226–230, 2006.

T. M. Worner, “Relative kindling effect of readmissions in alcoholics.,” *Alcohol & Alcoholism*, vol. 31, no. 4, pp. 375–380, 1996.

M. M. Yamada, M. Korman, and C. W. Hughes, “Predicting Rehospitalization of Persons with Severe Mental Illness.,” *J.Rehabil.*, vol. 66, no. 2, 2000.

C. Yeaman, J. Gambach, B. Bach, J. Manker, S. Diwan, and P. Corrigan, “What happens to people receiving inpatient psychiatric services in mixed rural and urban communities?.,” *Administration & Policy in Mental Health*, vol. 30, no. 3, pp. 247–253, 2003.

K. Yoshimasu, C. Kiyohara, and K. Ohkuma, “Efficacy of day care treatment against readmission in patients with schizophrenia: A comparison between out-patients with and without day care treatment.,” *Psychiatry & Clinical Neurosciences*, vol. 56, no. 4, pp. 397–401, 2002.

J. Yu and L. A. Warner, “Substance abuse treatment readmission patterns of Asian Americans: comparisons with other ethnic groups.,” *American Journal of Drug & Alcohol Abuse*, vol. 39, no. 1, pp. 23–27, 2013.

X. Yu, “Retrospective Study on Readmission of Psychiatric Patients,” *CHINESE MENTAL HEALTH JOURNAL*, vol. 16, no. 3, pp. 203–204, 2002.

A. D. Yussuf, S. A. Kuranga, O. R. Balogun, P. O. Ajiboye, B. A. Issa, O. Adegunloye, and M. T. Parakoyi, “Predictors of psychiatric readmissions to the psychiatric unit of a tertiary health facility in a Nigerian city—A 5-year study,” *African Journal of Psychiatry*, vol. 11, no. 3, pp. 187–190, 2008.

J. Zhang, C. Harvey, and C. Andrew, “Factors associated with length of stay and the risk of readmission in an acute psychiatric inpatient facility: a retrospective study.,” *Australian & New Zealand Journal of Psychiatry*, vol. 45, no. 7, pp. 578–585, 2011.

J. Zhang, C. Harvey, and C. Andrew, “Factors associated with the risk of readmission at an acute psychiatric inpatient facility: A retrospective cohort study,” *Australian and New Zealand journal of psychiatry*, vol. 42, pp. A120–A120, 2008.

Y. Zhou, Y. Ning, N. Fan, S. Mohamed, R. A. Rosenheck, and H. He, “Correlates of readmission risk and readmission days in a large psychiatric hospital in Guangzhou, China,” *Asia-Pacific Psychiatry*, vol. 6, no. 3, pp. 342–349, Sep. 2014.

N. Zilber, T. Hornik-Lurie, and Y. Lerner, “Predictors of early psychiatric rehospitalization: a national case register study.,” *Israel Journal of Psychiatry & Related Sciences*, vol. 48, no. 1, pp. 49–53, 2011.
