# Supplementary material for: Statistical methods and modelling techniques for analysing hospital readmission of discharged psychiatric patients: a systematic literature review
Source: BMC Psychiatry. 2016 Nov 18;16:413. doi: 10.1186/s12888-016-1128-7 (PMC5116202; doi:10.1186/s12888-016-1128-7)
Supplement: Additional file 4: — Additional information on cohort size and follow-up time of the reviewed studies: Contains tables with Quartiles, Minimum, Maximum and Mean of the patient sample sizes and follow-up time used in the reviewed studies. (DOCX 21 kb) [file 12888_2016_1128_MOESM4_ESM.docx]

**Additional file 4. Additional information on cohort size and follow-up time of the reviewed studies**

Number of patients in the cohorts analysed in the psychiatric readmission studies by the type of used method (years 1990-2014, N=407). In case patients are split into different sub-groups, for instance in case of looking at different diseases in parallel, the overall number of patients is used.

| **NUMBER OF PATIENTS– DISTRIBUTIONS** | **OVERALL** | **REGRESSION** | **NON-PARAMETRIC** | **PARAMETRIC** | **SURVIVAL** |
| --- | --- | --- | --- | --- | --- |
| Minimum | 7 | 30 | 10 | 7 | 30 |
| 1^st^ Quartile | 107 | 164 | 101 | 103 | 202 |
| Median | 306 | 417 | 202 | 233 | 615 |
| Mean | 6,357 | 8,590 | 4,635 | 4,713 | 10,720 |
| 3^rd^ Quartile | 1,231 | 1,972 | 819 | 686 | 3,404 |
| Maximum | 408,158 | 313,900 | 313,900 | 313,900 | 408,200 |
| Not available | 22 | 2 | 2 | 1 | 3 |

Follow-up time in months by the type of used method, reviewed studies on psychiatric readmission, years 1990–2014 (N=407).

| **FOLLOW-UP TIME– DISTRIBUTIONS** | **OVERALL** | **REGRESSION** | **NON-PARAMETRIC** | **PARAMETRIC** | **SURVIVAL** |
| --- | --- | --- | --- | --- | --- |
| Minimum | 0 | 0 | 0 | 0 | 1 |
| 1^st^ Quartile | 12 | 5.5 | 9.25 | 8 | 12 |
| Median | 12 | 12 | 12 | 12 | 21.5 |
| Mean | 33 | 25.58 | 32.98 | 29.47 | 38.56 |
| 3^rd^ Quartile | 36 | 24 | 36 | 36 | 36 |
| Maximum | 360 | 300 | 360 | 300 | 360 |
| Not available | 103 | 22 | 42 | 31 | 26 |
